# Spectrally Selective Absorbers/Emitters for Solar Steam Generation and Radiative Cooling‐Enabled Atmospheric Water Harvesting

**DOI:** 10.1002/gch2.202000058

**Published:** 2020-10-20

**Authors:** Yang Li, Chongjia Lin, Jingyuan Huang, Cheng Chi, Baoling Huang

**Affiliations:** ^1^ Department of Mechanical and Aerospace Engineering The Hong Kong University of Science and Technology Clear Water Bay Kowloon Hong Kong SAR 999077 China; ^2^ The Hong Kong University of Science and Technology Foshan Research Institute for Smart Manufacturing Clear Water Bay Kowloon Hong Kong SAR 999077 China

**Keywords:** atmospheric water harvesting, IR emitters, radiative cooling, selective absorbers, solar steam generation

## Abstract

Renewable energy harvesting from the sun and outer space have aroused significant interest over the past decades due to their great potential in addressing the energy crisis. Furthermore, the harvested renewable energy has benefited another global challenge, water scarcity. Both solar steam generation and passive radiative cooling‐enabled atmospheric water harvesting are promising technologies that produce freshwater in green and sustainable ways. Spectral control is extremely important to achieve high efficiency in the two complementary systems based on absorbing/emitting light in a specific wavelength range. For this reason, a broad variety of solar absorbers and IR emitters with great spectral selectivity have been developed. Although operating in different spectral regions, solar selective absorbers and IR selective emitters share similar design strategies. At this stage, it is urgent and necessary to review their progress and figure out their common optical characteristics. Herein, the fundamental mechanisms and recent progress in solar selective absorbers and IR selective emitters are summarized, and their applications in water production are reported. This review aims to identify the importance of selective absorbers/emitters and inspire more research works on selective absorbers/emitters through the summary of advances and the establishment of the connection between solar absorbers and IR emitters.

## Graphical abstract

Both solar steam generation and passive radiative cooling‐enabled atmospheric water harvesting are promising water production technologies powered by renewable energy harvesting from the sun and outer space. Spectral selectivity is crucial to both solar absorbers and infrared emitters in maximizing their efficiency. This review identifies the importance of selective absorbers/emitters and summarizes their advances, connections, and applications in freshwater production.

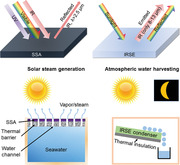

## 1 Introduction

According to the statistics reported by the United Nations, around 2.2 billion people, one‐third of the world population, are lacking access to safe freshwater and experiencing water scarcity.^[^ ^1^ ^]^ More startlingly, every day about 4400 children under 5 years old die from diseases caused by poor water sanitation around the world.^[^ ^2^ ^]^ The freshwater scarcity is becoming more and more severe because of the mismatch between the rising global demand and decreasing freshwater availability.^[^ ^3^ ^]^ The increasing demand for water mainly originates from four factors including population growth, improving living standards, increasing agricultural irrigation, and urbanization, while climate change and water waste are the two main reasons for the declining supply. Fortunately, the advances in science and engineering are offering more and more effective solutions, such as rainwater harvesting, seawater desalination, sewage recycling, and atmospheric water harvesting, to addressing this tough problem.^[^ ^4^ ^]^ However, most of these water production processes involve huge consumption of electricity and nonrenewable energy.^[^ ^5^ ^]^

Off‐grid water production technologies that use clean energy or even no energy are more attractive because they are environment‐friendly, energy‐saving, stable against power outages, and feasible for rural areas.^[^ ^5^, ^6^ ^]^ For instance, solar energy has been widely used to power the desalination process via thermal distillation.^[^ ^6^, ^7^ ^]^ In most of the conventional solar stills, black paint‐based solar absorbers are mounted on the bottom of the water tanks to harvest sunlight and heat the bulk water on top. More recently, a high‐efficiency distillation strategy, interfacial solar steam generation (ISSG), has been demonstrated to generate steam on the water‐air interface by using floating solar absorbers.^[^ ^8^, ^9^, ^10^, ^11^, ^12^, ^13^ ^]^ By the use of this strategy, heat is locally confined in solar absorbers that are thermally insulated from the bulk water to avoid enormous conductive heat losses. Solar absorbers, as key components in ISSG systems, are expected to possess the capability of effectively capturing the broad‐spectrum (0.3–2.5 µm) solar photons and converting them into heat. A broad variety of absorbers with near‐perfect (≈95%) and broadband solar absorption have been demonstrated, including carbon materials,^[^ ^8^, ^14^, ^15^, ^16^, ^17^, ^18^ ^]^ metal nanoparticles,^[^ ^9^, ^11^, ^12^, ^13^ ^]^ narrow bandgap semiconductors,^[^ ^19^, ^20^, ^21^, ^22^, ^23^, ^24^, ^25^, ^26^ ^]^ and black polymers.^[^ ^10^, ^27^, ^28^ ^]^ According to Kirchhoff's law, in thermodynamic equilibrium, emission at a certain wavelength is equal to the absorption due to reciprocity.^[^ ^29^ ^]^ Therefore, ideally, a solar absorber should show no absorption (emission) in the IR region (>2.5 µm) to suppress radiative heat losses and achieve high solar‐thermal efficiency, which is more challenging.^[^ ^11^, ^30^, ^31^, ^32^ ^]^ Without the use of such spectrally selective absorbers, it is difficult or even impossible to generate high‐temperature steam under the illumination of one sun for many specific applications, such as water boiling (>100 °C) and sterilization (>121 °C).^[^ ^8^, ^33^, ^34^ ^]^ Efficient solar selective absorbers (SSAs) are rare in natural materials and are usually realized by constructing photonic metamaterials/metasurfaces.

Atmospheric water harvesting (AWH) that harvests water from the ambient air is another promising off‐grid technology.^[^ ^35^, ^36^, ^37^, ^38^ ^]^ At any given time in the atmosphere, there is tremendous amounts of water (1.3 × 10^22^ L) existing as vapor and droplet, which is a natural resource for freshwater production.^[^ ^39^ ^]^ One kind of AWH device uses porous materials such as metal–organic frameworks (MOF) to adsorb vapor in humid air, which can be released by solar heating.^[^ ^39^, ^40^, ^41^, ^42^ ^]^ Passive atmospheric water harvesting (PAWH) can even utilize the natural temperature drop to cool the air below its dew point and extract freshwater, requiring no external energy sources and vapor‐adsorption materials.^[^ ^43^, ^44^ ^]^ In fact, the PAWH process is inspired by the dewing phenomenon at night, which is caused by the notable temperature drop through radiating heat to the extremely cold outer space (≈3 K), known as passive radiative cooling.^[^ ^45^, ^46^ ^]^ Therefore, PAWH enabled by radiative cooling is a complementary technology to solar steam generation. It can produce freshwater in those inland and desert regions where bulk water for steam generation is scarce. Moreover, PAWH is especially efficient at night when sunlight is absent. For the sake of superior cooling performance, a wide variety of radiative coolers have been demonstrated to embrace strong IR emission in the atmospheric window of 8–13 µm.^[^ ^46^, ^47^, ^48^, ^49^, ^50^, ^51^, ^52^ ^]^ Ideal radiative coolers should also show reduced emission in other IR regions beyond the atmospheric window to avoid unwanted parasitic heat gain from the environment, which are extremely important for achieving large temperature drops.^[^ ^46^, ^52^, ^53^ ^]^

Spectrally selective absorbers/emitters enabled by photonic metasurfaces are advantageous both in the solar‐thermal conversion and in the passive radiative cooling systems, both of which are core technologies in the two complementary off‐grid water production approaches. Over the past decades, great advances have been achieved in the area of selective absorbers/emitters for specific wavelength regions, from UV (<0.4 µm), visible (0.4–0.7 µm), near‐IR (0.7–2.5 µm), mid‐wavelength IR (MWIR, 3–8 µm), and long‐wavelength IR (LWIR, 8–14 µm). In this review, at first, we will briefly discuss the criteria for efficient ISSG and AWH. Then, the fundamental mechanism and recent progress in the development of SSAs and IR selective emitters (IRSEs) will be summarized, and holistic connections between SSAs and IRSEs will be established. Also, current applications of SSAs and IRSEs on ISSG and AWH will be reviewed. Last, a conclusion and outlook of SSAs and IRSEs will be discussed.

## 2 Selective Absorbers (SAs) and Selective Emitters (SEs)

**Figure 1:**
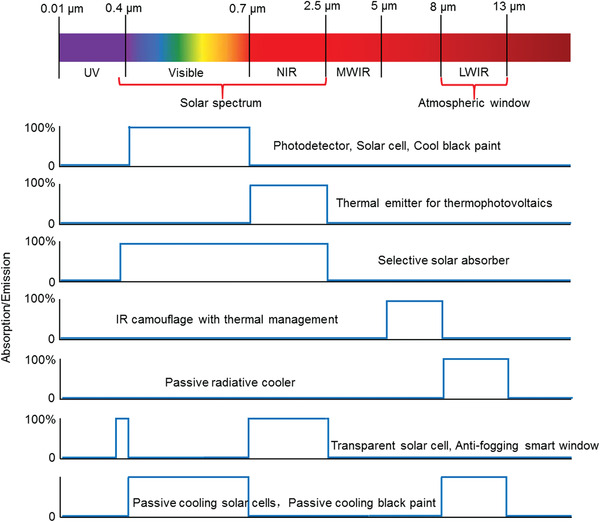
Spectrally selective absorbers/emitters SAs (SEs) with perfect absorption/emission for specific wavelength regions in the UV–vis–IR range, and their potential applications.

Spectral selectivity refers to the ability of a surface or film to selectively absorb or transmit the desirable light, and at the same time, reflect the undesirable light.^[^ ^30^ ^]^ Particularly, selective absorption (emission) is attractive in a lot of fields, such as solar energy conversion,^[^ ^30^, ^54^ ^]^ thermal management,^[^ ^55^, ^56^ ^]^ radiative cooling,^[^ ^46^ ^]^ and smart windows.^[^ ^57^, ^58^ ^]^ **Figure** **1** summarizes seven classes of SAs (SEs) targeting at different wavelength regions and their applications. The first class of selective absorber for the visible light (0.4–0.7 µm) has been widely used in photodetectors and solar cells.^[^ ^59^, ^60^ ^]^ Another potential application is cool black paints for cars and buildings because they only absorb the visible light but reflect sunlight in the near‐IR region, and therefore show relatively lower temperatures compared to conventional black paints.^[^ ^61^ ^]^ The second class of selective emitter (absorber) in the NIR region (0.7–2.5 µm) is extremely desired in thermophotovoltaic devices^[^ ^62^, ^63^, ^64^ ^]^ and transparent NIR solar cells.^[^ ^65^ ^]^ The third class of selective absorber is the well‐known SSA that can perfectly harvest the full‐spectrum sunlight and reflect the IR light to avoid thermal radiation heat loss, which will be discussed in this review in detail.^[^ ^30^, ^66^ ^]^ The fourth class of selective emitter can emit light only in the wavelength range of 5–8 µm, showing great promise in IR camouflage and radiative cooling of high‐temperature objects.^[^ ^67^, ^68^ ^]^ The fifth class of selective emitter refers to passive radiative coolers that selectively emit thermal radiation to the remote cool sky (≈3 K) through the atmospheric window (8–13 µm, LWIR region), named as IRSE, which is another emphasis in this review.^[^ ^46^, ^55^ ^]^ The sixth class of selective absorber is a dual‐band solar absorber with strong absorption in both the UV and NIR regions, which is also desired in transparent solar cells.^[^ ^58^, ^69^, ^70^ ^]^ The seventh class of selective absorber (emitter) is also a dual‐band absorber that has high visible light absorption and high LWIR emission. Potential applications of such an absorber include passive radiative cooling enhanced solar cells,^[^ ^71^ ^]^ and low‐temperature passive cooling black paints. By combining single‐band SAs (SEs) with each other, more classes of dual‐band and even multiband SAs (SEs) can be developed for specific applications. Despite great advances, there lacks a holistic connection for selective absorber/emitters operating in different spectra, which is also one of the motivations of this review.

**Figure 2:**
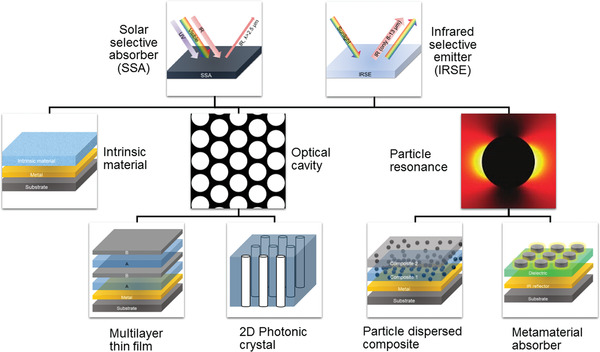
Classification of current SAs (SEs) according to their absorption mechanisms: intrinsic materials, optical cavities, and particle‐based resonance.

Such smart surfaces or films with great spectral selectivity are realized by two approaches: intrinsic materials and photonic metamaterials/metasurfaces, as classified in **Figure** **2**. A few intrinsic materials exhibit strong and selective light absorption in some wavelengths, such as TiO_2_ for UV light, Si and Ge for visible light, and SiO_2_, Si_3_N_4_, and HfO_2_ for LWIR light. However, the spectral selectivity of intrinsic materials is limited and the number of these materials is insufficient, so most SAs (SEs) are realized by artificially designed photonic nanostructures, known as photonic metamaterials/metasurfaces. Generally, the photonic metasurfaces can be regarded as effective electromagnetic mediums in the interaction with light. Photonic metasurface enabled SAs (SEs) with near‐perfect absorption in various spectral regions share the same optical principle in structural design. The effective impedance of the metasurfaces *Z*

(1) $$\begin{array}{c} Z=\sqrt{\frac{\mu }{\varepsilon }}=\sqrt{\frac{\mu_{0}\mu_{\text{r}}}{\varepsilon_{0}\varepsilon_{\text{r}}}}=Z_{0}\sqrt{\frac{\mu_{\text{r}}}{\varepsilon_{\text{r}}}}=Z_{0}\frac{\mu_{\text{r}}}{n} \end{array}$$

has to match that of the free space (*Z*_0_) in the target wavelength range to eliminate reflection (*R*), where μ (μ_0_) and ε (ε_0_) are the permittivity of and permeability of the metasurfaces (free space), respectively. μ_r_, ε_r_, and *n* are the relative permittivity, permeability, and refractive index of the metasurfaces. The reflection of the metasurfaces under normal incidence is then^[^ ^72^ ^]^

(2) $$\begin{array}{c} R={\left|\frac{Z-Z_{0}}{Z+Z_{0}}\right|}^{2}=0 \end{array}$$

Typically, a metallic reflective film is used as the ground layer, which is optically thick to ensure zero transmission (*T*). Consequently, the absorption *A* = 1 – *R* – *T* reaches 100%.

To achieve impedance match and therefore near‐perfect absorption/emission, a broad variety of strategies have been utilized to develop such photonic metasurfaces for different spectra. We classified these reported photonic metasurfaces operating in different spectral regions into two categories according to their absorption mechanisms: optical cavity and particle‐based resonance. Optical cavities refer to the multilayer thin films^[^ ^46^, ^66^ ^]^ and photonic crystals^[^ ^73^ ^]^ that trap light by cavity resonance, while particle‐based resonance metasurfaces include the metal–dielectric–metal perfect metamaterial absorbers^[^ ^74^, ^75^ ^]^ and the particle‐dispersed composites.^[^ ^30^, ^47^ ^]^

## 3 Criteria for Efficient Solar Steam Generation and Atmospheric Water Harvesting

### 3.1 Criteria for Efficient Solar Steam Generation

Solar steam generation is a sustainable technology that converts the abundant and clean solar energy into thermal energy, which is utilized to heat water and generate steam. This is a typical thermodynamic process that must follow the first thermodynamic law of energy conservation. The solar‐thermal energy conversion efficiency is the ratio of net absorbed heat to the total incident solar energy.^[^ ^14^, ^34^ ^]^ First of all, to achieve high efficiency, the incident solar energy that covers a broad spectrum from 0.3 to 2.5 µm should be absorbed as much as possible. Suppressing heat losses to the bulk water and environment is as important as achieving high solar absorption to realize efficient solar steam generation systems.^[^ ^8^ ^]^ There are three paths of heat loss: conductive heat loss to the bulk water *q*_cond_, convective heat loss to the environment air *q*_conv_, and radiative heat loss to the space *q*_rad_. The energy conversion efficiency of solar steam generation systems is limited by^[^ ^14^, ^34^ ^]^

(3) $$\begin{array}{c} \eta =\left(\bar{\alpha }-\frac{q_{\text{cond}}+q_{\text{conv}}+q_{\text{rad}}}{Cq_{\text{solar}}}\right)\times 100\% \end{array}$$

where *q*_cond_, *q*_conv_, and *q*_rad_ are the conductive, convective, and radiative heat losses, respectively. $\bar{\alpha }$, *q*_solar_ and *C* are the spectrally averaged solar absorptance, incident solar flux (AM 1.5G, 1 kW m^−2^), and the optical concentration ratio, respectively. For these solar evaporation devices, the solar‐thermal efficiency is also defined as^[^ ^8^ ^]^

(4) $$\begin{array}{c} \eta =\frac{\dot{m}h_{\text{LV}}}{Cq_{\text{solar}}}\times 100\% \end{array}$$

where $\dot{m}$ is the evaporation rate and *h*_LV_ is the total enthalpy of the liquid–vapor phase change including both sensible heat and phase‐change enthalpy. The result of Equations (3) and (4) should be consistent.

#### 3.1.1 Conductive Heat Loss Management

**Figure 3:**
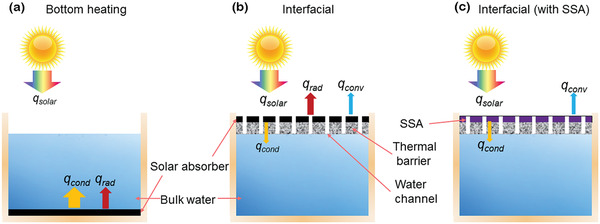
Schematic of a) bottom heating solar steam generation, b) interfacial solar steam generation (ISSG) with a blackbody absorber and c) an SSA. *q*_solar_, *q*_cond_, *q*_conv_, and *q*_rad_ represent solar radiation power, conductive, convective, and radiative heat losses, respectively.

Conductive heat loss to water can be estimated by the 1D conductive model^[^ ^14^, ^34^ ^]^

(5) $$\begin{array}{c} q_{\text{cond}}=-k\frac{T_{0}-T_{\text{A}}}{l} \end{array}$$

where *T*_0_ is the water and air temperature, *T*_A_ is the absorber temperature, *k* is the thermal conductivity of water, and *l* is the heat transfer length in water. To reduce the heat conduction from solar absorbers to bulk water, interfacial solar steam generation (ISSG) has been demonstrated to separate solar absorbers from the bulk water by porous thermal barriers with low effective thermal conductivity, as shown in **Figure** **3**.^[^ ^8^, ^9^, ^10^, ^11^, ^12^, ^13^ ^]^ The water is pumped to solar absorbers by the capillary effect of the porous structures or additionally adopted water channels. Meanwhile, the underneath bulk water is not heated up and therefore heat can be localized at the interface of solar absorbers and the pumped water in a limited area. As a result, compared to the conventional solar steam generation, solar‐thermal energy conversion efficiency has been improved from 30–40% to >80%.^[^ ^16^, ^18^, ^23^, ^25^, ^28^ ^]^

#### 3.1.2 Convective Heat Loss Management

Heat convection with the environment is another route of energy loss in ISSG. The convective heat loss from the absorber to the environment can be calculated from Newton's cooling law^[^ ^14^, ^34^ ^]^

(6) $$\begin{array}{c} q_{\text{conv}}=\;h\left(T_{\text{A}}-T_{0}\right) \end{array}$$

where *h* is the convective heat transfer coefficient. To suppress the convective loss, the *h* (natural convective coefficient, 5–10 W m^−2^ K^−1^) must be reduced to a very small value. The most effective method of reducing the *h* is enclosing the device in a closed space and pumping it to a vacuum condition.^[^ ^32^, ^33^, ^76^ ^]^ In fact, vacuum tubes are widely used in conventional solar heating panels and parabolic trough collectors.^[^ ^32^, ^76^ ^]^ Many low‐cost strategies can also be applied to suppress the convective loss, such as creating narrow air gaps on top of the solar absorber like insulating windows with double‐layer glass.^[^ ^34^, ^77^ ^]^

#### 3.1.3 Radiative Heat Loss Management

All materials with a temperature higher than 0 K will emit thermal radiation as the form of electromagnetic waves caused by the movements of particles in materials.^[^ ^29^ ^]^ The temperature of materials determines the wavelength distribution of their thermal radiation. For materials with a temperature near 25 °C, the thermal radiation mainly locates in the LWIR region, so thermal radiation is also called IR radiation in many areas. The ratio of the radiation of any material to that of a black body at the same temperature is emissivity. Radiative heat loss of solar absorber *q*_rad_ is given by^[^ ^14^, ^34^ ^]^

(7) $$\begin{array}{c} q_{\text{rad}}=\bar{\varepsilon }\sigma \left(T_{\text{A}}^{4}-T_{0}^{4}\right) \end{array}$$

where $\bar{\varepsilon }$ and σ are the spectrally averaged emissivity of solar absorbers and Stefan–Boltzmann constant. As we mentioned above, the emission at any wavelength is equal to the absorption,^[^ ^29^ ^]^ therefore, the $\bar{\varepsilon }$ can be calculated from the IR absorption spectrum measured by Fourier‐transform infrared spectroscopy. By using IR cameras, the $\bar{\varepsilon }$ of a sample with a known surface temperature can also be roughly derived.

**Figure 4:**
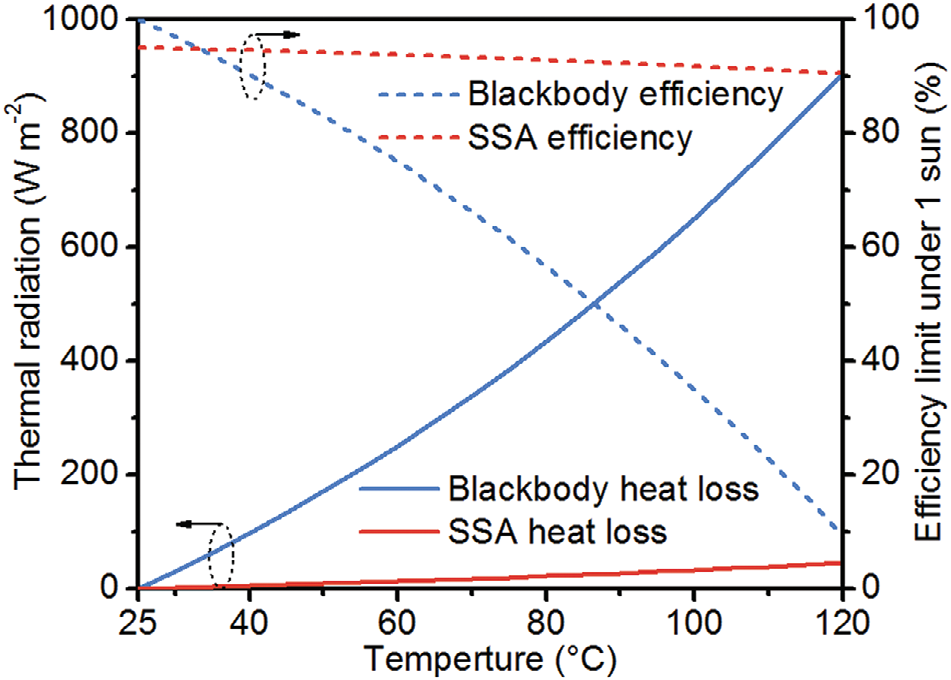
Radiative heat losses of a blackbody absorber and a commercial SSA ($\bar{\alpha }=95\%$, $\varepsilon_{100{\;}^{\circ }\text{C}}=5\%$) as a function of operating temperature, and the corresponding solar‐thermal conversion efficiency limit under the illumination of 1 sun (1 kW m^−2^).

**Figure** **4** shows the radiative heat losses of a black body absorber ($\bar{\alpha }$ = $\bar{\varepsilon }$ *=* 100%) and a commercial SSA ($\bar{\alpha }$ = 95%,$\bar{\varepsilon }$ = 5%) when the ambient temperature is 25 °C. For reported solar absorbers in ISSG systems under the illumination of one sun (AM 1.5G, 1000 W m^−2^), the surface temperature of most of them is around 35–45 °C.^[^ ^8^, ^9^, ^10^, ^11^, ^14^, ^16^, ^28^, ^78^ ^]^ The radiative heat loss is around 63 to 133 W m^−2^, corresponding to the theoretical maximum solar thermal efficiency of 93.7% and 86.7% under 1 sun. Moreover, radiative heat loss increases more rapidly with the absorber temperature than conductive and convective losses due to the fourth power dependence. At the water boiling point of 100 °C, the thermal radiation power of the black body absorber is as high as 650 W m^−2^ (ambient temperature, 25 °C), accounting for 65% of 1 sun power. Considering the increasing conductive and convective heat transfer at higher temperatures together, it is difficult to produce steam of >100 °C under unconcentrated solar radiation (≤1 sun) in ambient air. As a result, the generation of such high‐temperature steam generally requires a high optical concentration of >10.^[^ ^8^, ^33^, ^34^ ^]^ However, both optical concentrators (200 USD m^−2^) and solar tracking accessories are far more expensive than the steam generation systems.^[^ ^32^, ^34^ ^]^ For the concerns from both efficiency and cost, reducing heat loss from thermal radiation is critical to ISSG systems, especially those high‐temperature systems. Moreover, it can be readily realized by the utilization of low‐emissivity solar absorbers, i.e., SSAs.^[^ ^34^, ^79^, ^80^ ^]^ The 1 sun efficiency limit of commercial SSAs at 100 °C (91.8%) is much higher than that of blackbody absorbers (35.0%). In 2016, a commercial SSA was introduced to the ISSG systems by Chen's group that demonstrated the first ISSG device. Accompanied by thermal concentration, 100 °C steam generation in ambient air was produced under solar flux even below 1 kW m^−2^.^[^ ^34^ ^]^

### 3.2 Criteria for Efficient AWH

Solar steam generation is effective in producing large amounts of freshwater from seawater, rainwater, and wastewater. However, for those inland and desert areas, the lack of bulk water hinders the wide use of solar distillation, so plenty of vapor and droplet in the air provides another solution. Water from the air can condense on a surface with a temperature lower than its dew point.^[^ ^43^, ^44^ ^]^ The condensing rate of AWH can be analyzed in terms of power at a certain required temperature. Here we can calculate the condensing rate of AWH according to

(8) $$\begin{array}{c} \dot{m}=\frac{P_{\text{cooling}}(T_{\text{d}.\text{p}.})-P_{\text{parasitic}}}{L(T_{\text{d}.\text{p}.})+C(T_{\text{amb}}-T_{\text{d}.\text{p}.})} \end{array}$$

where *P*_cool_ is the cooling power, *P*_parasitic_ is the parasitic heat gain by the device, *T*_d.p._ is the dew point temperature, *T*_amb_ is the ambient air temperature, *C* is the specific heat capacity of water, and *L* is the specific latent heat. The two concerns, cooling temperature and cooling power answer two questions, respectively: can the device condense water and how much? Specifically, the condensing rate depends on i) whether the temperature of the condenser can be less than the required dew point of the vapor, and ii) the value of effective power in the device to decrease the vapor's temperature and release its latent heat.

#### 3.2.1 Cooling Temperature to Reach the Dew Point

**Figure 5:**
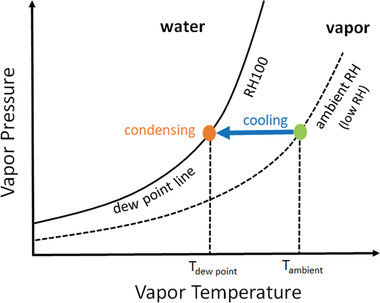
Processes of cooling and condensing for atmospheric water harvesting.

Cooling is a necessary process for AWH. As shown in the phase diagram of water in **Figure** **5**, in the initial state, the near‐ground atmosphere has a low relative humidity (RH). With a given vapor pressure or so‐called “absolute humidity,” in order to make condensation happen, RH of the vapor needs to be increased until it reaches the dew point line where RH is saturated. As a result, a cooling source for the atmosphere vapor to change from the ambient temperature to the corresponding required dew point temperature is necessary. The cooling source can be a refrigerator system or an off‐grid radiative cooler.^[^ ^45^, ^81^ ^]^

**Figure 6:**
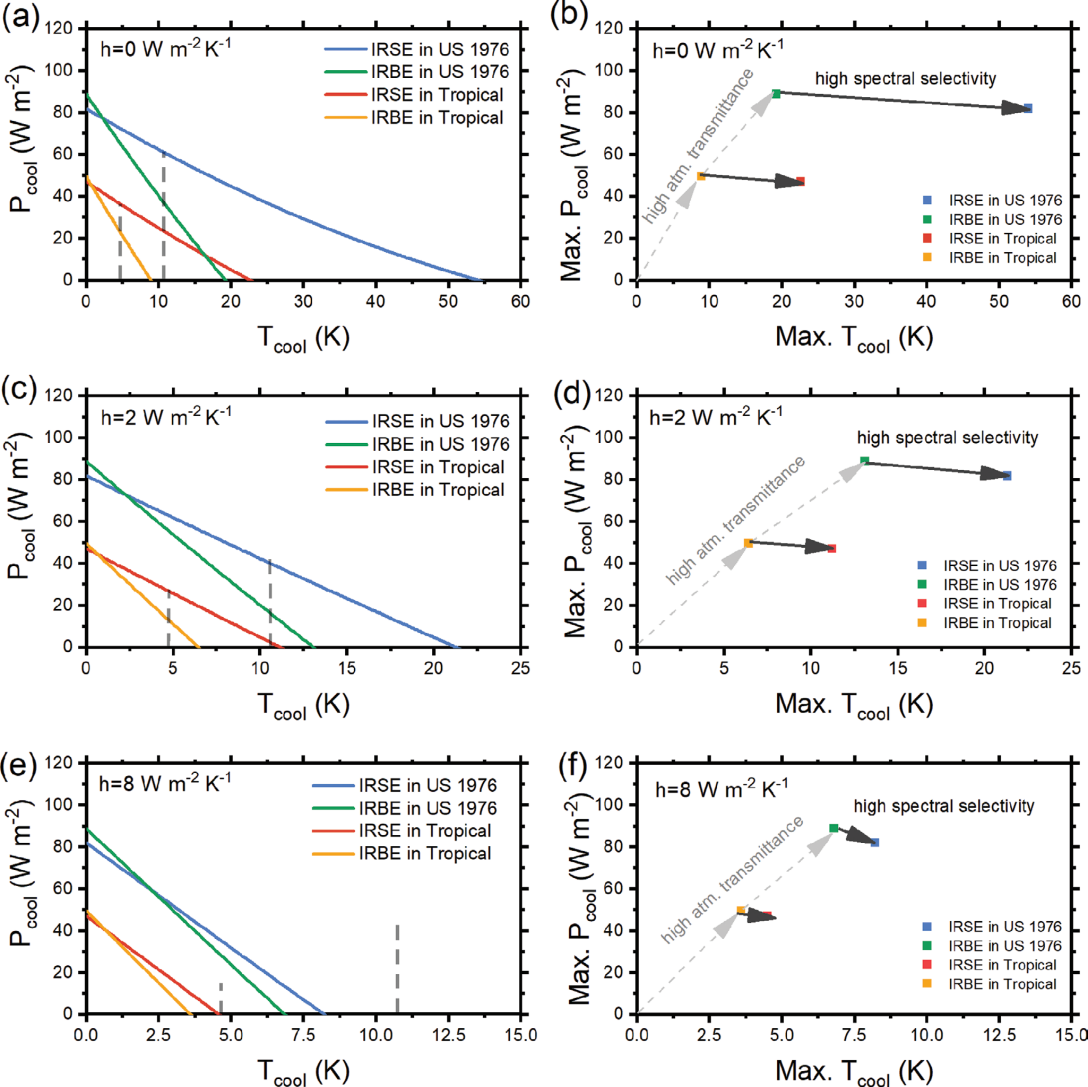
Cooling performance of ideal IR selective emitters (IRSE) and IR broadband emitters (IRBE) in different environments. a,c,e) The cooling process at the dew point, with convective coefficient *h* = 0, 2, 8 W m^−2^ K^−1^. b,d,f) Internal factor and external factor for cooling performance, showing two trends for maximum cooling power and maximum cooling temperature, with convective coefficient *h* = 0, 2, 8 W m^−2^ K^−1^. Referred to the database of typical climate modes provided by MODTRAN 6, in this calculation, *T*_amb_ = 288.15 K for US Standard 1976 and 299.70 K for Tropical. Critical *T*_cool_ for reaching *T*_d.p_ *_._* is 10.68 K for US Standard 1976, and 4.69 K for Tropical, which are indicated in dash lines in (a), (c), and (e).

The cooling temperature is defined as the ambient air temperature minus the temperature of the radiative emitter. In the radiative cooling technology, the cooling temperature is affected by external factors including atmosphere conditions (e.g., air temperature, humidity, cloud, etc.), solar irradiation, and wind speed. However, for given external environments, the spectral selectivity of the radiative emitter becomes a dominant factor for its cooling performance.^[^ ^46^, ^82^, ^83^ ^]^ As shown in **Figure** **6**a, the cooling process starts from the initial point of minimum cooling temperature and maximum cooling power and will reach equilibrium at its maximum cooling temperature with zero cooling power or at *T*_cool_ = *T*_amb_–*T*_d.p._, whichever occurs earlier.

An ideal IR broadband emitter (IRBE) has unity emissivity over the entire IR range of 2.5–30 µm and zero emissivity elsewhere; while an ideal IRSE has unity emissivity only at 8–13 µm and zero emissivity elsewhere. As shown in Figure 6b, under the same ambient condition, the emitter with higher selectivity offers a much higher maximum cooling temperature while its maximum cooling power sacrifices a little. For a given environment condition, higher selectivity leads to a higher cooling temperature due to the suppression of parasitic heating, thus more likely to reach the required cooling temperature for condensation, especially for those areas with a thick atmosphere and considerable parasitic heat gain.

#### 3.2.2 Effective Power for Cooling and Condensing

As shown in Equation (8), the AWH condensing rate is strongly dependent on the effective cooling, which is defined as the cooling power provided by the emitter (with two examples as shown in Figure 6a), minus the parasitic heat gain from the environment.^[^ ^46^ ^]^ And it is used to complete the processes of reducing the temperature of vapor from ambient temperature to dew point and absorbing the water's latent heat during the phase change.

Similar to the discussion in Section 3.1, the parasitic heat gain for AWH also consists of conductive heat, convective heat, and radiative heat. As discussed in the Supporting Information of a previous work,^[^ ^52^ ^]^ there are several approaches to minimize parasitic heat gain in radiative cooling devices: optimizing thermal contact to minimize conduction, using a high vacuum to eliminate air conduction and convection, using radiation shields at the backside of the selective emitter to reduce the radiation. Radiative condenser device design can be optimized by two approaches: using the top surface of the emitter for cooling and the bottom surface for condensing, and using a pump to control the fresh airspeed under the emitter.^[^ ^84^ ^]^

For radiative cooling, the convection coefficient *h* is generally considered as 8 W m^−2^ K^−1^ for typical rooftop setups.^[^ ^84^ ^]^ It can be as much as 6.9 or 7.3 W m^−2^ K^−1^ for nonvacuum polyethylene‐sealed devices,^[^ ^46^, ^85^ ^]^ and can be minimized to 0.2–0.3 W m^−2^ K^−1^ for vacuum designs.^[^ ^52^ ^]^ The convection coefficient is case‐specific, and setups in the open air are largely affected by natural convection. There were some studies correlated *h* to wind speeds.^[^ ^46^, ^86^, ^87^, ^88^, ^89^ ^]^ And for radiative condensing, *h* is considered as 2 W m^−2^ K^−1^ for optimized setups. While Figure 6a,b shows an ideal situation with *h* = 0 W m^−2^ K^−1^, Figure 6c–f provides estimation in practical conditions with *h* = 2 and 8 W m^−2^ K^−1^. It can be seen that both IRSE and IRBE are able to condense at typical modes of US Standard 1976 and Tropical when *h* = 2 W m^−2^ K^−1^, but they cannot condense when *h* = 8 W m^−2^ K^−1^.

Surface wettability of emitters and condensing panels is another key issue related to effective condensing, but there is still a lack of sufficient studies on this part. We believe that emitters with hydrophobic surfaces are more likely to minimize the parasitic gain from the water film, while a proper level of wettability (or combination of hydrophobic and hydrophilic by microchannel design) may be beneficial to the dropwise condensation.

#### 3.2.3 Selective IR Emitters for Atmospheric Water Harvesting

**Figure 7:**
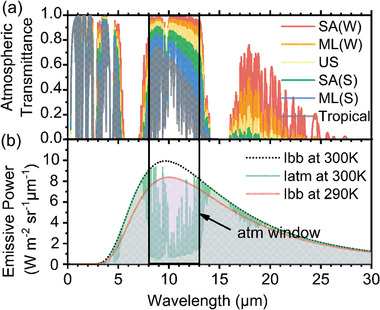
Atmospheric window and radiative cooling mechanism. a) Atmosphere transmittance obtained from MODTRAN 6 with SA(W), ML(W), US 1976, SA(S), ML(S) representing for subarctic winter, middle‐latitude winter, US standard 1976, subarctic summer, and middle latitude summer, respectively. b) The importance of spectral selectivity at the atmospheric window for the radiative cooling mechanism, with Ibb and Iatm representing for blackbody radiation and radiation from the atmosphere at US standard 1976, respectively. (Please note that, for simplicity, emissive power illustrated here is only for normal direction.)

In this section, the significance of emitters’ spectral selectivity for AWH will be discussed. Radiative cooling works by emitting thermal energy from the emitter to the extremely cold outer space (≈3 K) through atmospheric windows that have high transmittance. As shown in **Figure** **7**a, there are several atmospheric windows including 3–5, 8–13, and 16–22 µm. Such spectral‐dependent transmittance is due to the absorption phenomenon of waves caused by atmospheric molecules’ movements, and it is ever‐changing with the atmospheric conditions. As the radiation peak of a 300 K black body locates at around 10 µm, 8–13 µm is defined as the main atmospheric window in radiative cooling.

As shown in Figure 7b, taking the US standard 1976 atmosphere as an instance, when an emitter emits radiation at 290 K (indicated by the red curve), it also receives radiation from the atmosphere at 300 K (indicated by the green curve). We can see that, inside the atmospheric window, the outgoing radiation is larger than the incoming radiation within the atmospheric window, positively contributing to radiative cooling. But the situation is opposite outside the atmospheric window. That is why radiative coolers are always designed to possess as high emissivity as possible within the atmospheric window, but as low emissivity as possible outside the atmospheric window.

As examples shown in Figure 6b,d,f, for a given external environment and parasitic heat gain condition, a higher spectral selectivity leads to a larger maximum cooling temperature at its equilibrium state, so it is easier to reach the critical *T*_cool_ for condensing, where critical *T*_cool_ = *T*_amb_–*T*_d.p._. As shown in Figure 6a,c,e, the spectral selectivity also determines whether and how much cooling power can be successfully obtained to fulfill the energy requirement for water harvesting.

## 4 Mechanism and Recent Progress of SSAs

**Figure 8:**
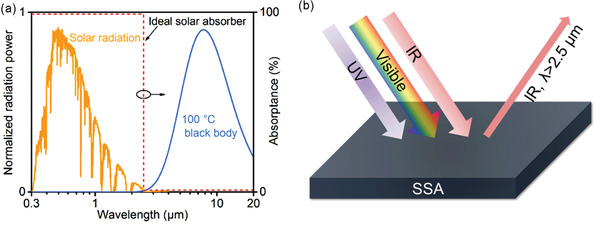
a) AM 1.5G solar spectrum, 100 °C black body radiation spectrum, and the corresponding absorption spectrum of an ideal solar absorber with a cut‐off wavelength at 2.5 µm. The cut‐off wavelength strongly depends on the operating temperature *T*_A_ and the solar concentration ratio *C*. b) Schematic of spectrally solar selective absorbers (SSAs) with perfect absorption in the solar spectrum (0.3–2.5 µm) and no absorption in the mid‐IR region (>2.5 µm).

SSAs are of great importance for solar‐thermal conversion systems to achieve maximum solar‐thermal efficiency including solar heating, concentrated solar powers,^[^ ^32^, ^66^ ^]^ steam generation,^[^ ^33^, ^34^ ^]^ deicing,^[^ ^90^ ^]^ and photothermal catalysis.^[^ ^91^ ^]^ **Figure** **8** shows the normalized solar radiation spectrum and 100 °C blackbody radiation spectrum, intersecting at 2.5 µm. In other words, the cut‐off wavelength of an ideal SSA with a step‐function spectrum should be 2.5 µm for the target applications at 100 °C under 1 sun. The cut‐off wavelength is a critical parameter in the design of SSAs, which highly relies on both the solar concentration ratio *C* and the operating temperature *T*_A_. Ideal step‐function spectra are difficult to achieve in real SSAs, so the cut‐off should locate in the wavelength where the absorption (emission) intensity is around 50%. High *C* and low *T*_A_ correspond to longer cut‐off wavelengths.^[^ ^30^, ^32^, ^74^ ^]^ The optical performance of SSAs is characterized by the efficiency of their solar‐thermal conversion processes, which is simply defined as^[^ ^32^, ^74^ ^]^

(9) $$\begin{array}{c} \eta_{\text{solar}-\text{th}}=\bar{\alpha }-\bar{\varepsilon }\frac{\sigma \left(T_{\text{A}}^{4}-T_{0}^{4}\right)}{Cq_{\text{solar}}} \end{array}$$

Equation (9) is a special case in Equation (1) when convective and conductive heat losses are neglected.

The $\bar{\alpha }$ and $\bar{\varepsilon }$ are defined as

(10) $$\begin{array}{c} \bar{\alpha }=\frac{\int_{0.3\;\mu \text{m}}^{4\;\mu \text{m}}\text{d}\lambda \cdot \alpha (\lambda )\cdot E_{\text{solar}}(\lambda )}{q_{\text{solar}}} \end{array}$$

(11) $$\begin{array}{c} \bar{\varepsilon }=\frac{\int_{0}^{20\;\mu \text{m}}\text{d}\lambda \cdot \varepsilon (\lambda )\cdot E_{\text{B}}(\lambda ,T_{\text{A}})}{\sigma T_{\text{A}}{}^{4}} \end{array}$$

where *E*_solar_(λ) and *E*_B_(*λ,T*_A_) represent the spectral solar power (AM 1.5G) and blackbody emission, respectively. *Α*(λ) and ε(λ) are the spectral absorptance and emissivity at the wavelength λ.

Besides the great spectral selectivity, ideal SSAs are desired to possess more appealing advantages, such as excellent thermal and chemical stability, scalability, and low cost. Over the past several decades, great efforts have been made in the development of SSAs. Generally, SSAs were classified into six classes in other reviews according to their configurations,^[^ ^30^, ^32^, ^92^, ^93^ ^]^ including intrinsic absorbers, ceramic–metal composites (cermets), semiconductor–metal tandems, multilayered thin films, plasmonic metamaterial absorbers (PMAs), and photonic crystals (PhCs). Intrinsic absorbers and semiconductor–metal tandems correspond to the intrinsic materials categorized in this review (Figure 2). Multilayer thin films and PhCs correspond to optical cavity‐enabled SSAs. PMAs and cermets correspond to particle resonance‐enabled SSAs due to the excitation of plasmon resonance of metal nanoparticles. Here we will mainly focus on those recently reported SSAs with high overall performance.

### 4.1 Intrinsic SSAs

A few ceramics, such as transitional metal carbides, borides, and nitrides (TiC, ZrC, HfC, TiN, ZrN, TiB_2_, ZrB_2_, etc.), and semiconductors (Si, Ge, and SiGe) intrinsically show high solar absorption due to the interband or intraband transition.^[^ ^94^, ^95^, ^96^, ^97^ ^]^ For instance, it is well known that semiconductors can selectively absorb the photons with energy above their bandgaps and are transparent to those photons with energy below the bandgaps, exhibiting inherent spectral selectivity. Based on this fact, some SSAs were developed using these materials as absorbing medium coated on a metallic reflector. Gao et al. reported a stainless steel/ZrB_2_/Al_2_O_3_ SSA deposited by magnetron sputtering, exhibiting great spectral selectivity ($\bar{\alpha }$ = 92%, $\bar{\varepsilon }$ = 11% at 82 °C) and decent thermal stability at 500 °C in vacuum.^[^ ^98^ ^]^ Among semiconductors, amorphous Si is the first material that was used to construct SSAs with an $\bar{\alpha }$ of ≈80% and an $\bar{\varepsilon }$ of ≈13% at 400 °C.^[^ ^95^ ^]^ A Ge‐based multilayer semiconductor absorber that with a measured $\bar{\alpha }$ of 76% and a low $\bar{\varepsilon }$ of 5% was presented by Thomas et al.^[^ ^99^ ^]^ A multiscaled SiGe‐based SSA with nanostructures was developed to show an improved $\bar{\alpha }$ of ≈90–95% and an $\bar{\varepsilon }$ of ≈30% at 100 °C.^[^ ^100^ ^]^ In short, SSAs made of intrinsic materials have a simple structure, but their spectral selectivity, solar absorption, and thermal stability are far from satisfactory because few intrinsic materials have highly selective and near‐perfect solar absorption. By adopting these intrinsic materials as components in multilayer thin‐film absorbers, high‐performance SSAs can be realized.

### 4.2 Optical Cavity‐Enabled SSAs

Optical cavities refer to nanostructures that have the capability of confining the light by multiple reflections and forming resonators due to the effects of interference. Only light with certain wavelengths will be supported in the optical resonator, indicating spectral selectivity. Near‐perfect light absorption is achieved in lossy component materials after multiple reflections.^[^ ^54^, ^101^ ^]^ The cut‐off wavelength of optical resonators strongly depends on the cavity size. Both multilayer metal–dielectric films and PhCs are optical resonators: one falls into 1D optical resonators, and the other falls into 2D and 3D resonators.

#### 4.2.1 Multilayer Thin Films

**Figure 9:**
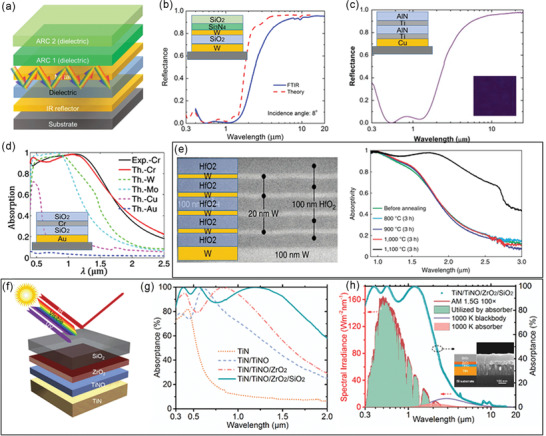
SSAs based on multilayer thin films. a) Schematic of SSAs based on multilayer metal–dielectric thin films. b) Measured and simulated reflectance spectra of a W/SiO_2_/W/Si_3_N_4_/SiO_2_ SSA. Reproduced with permission.^[^ ^102^ ^]^ Copyright 2018, Elsevier. c) Reflectance spectrum of a Cu/Ti/AlN/Ti/AlN SSA. Reproduced with permission.^[^ ^107^ ^]^ Copyright 2013, Elsevier. d) Measured and simulated absorptance spectra of Au/SiO_2_/Cr (W, Mo, Cu, and Au)/SiO_2_ SSAs. Reproduced with permission.^[^ ^106^ ^]^ Copyright 2015, Optical Society of America. e) Schematic, TEM image, and absorptance spectra of a ten‐layer W–HfO_2_ SSA. Reproduced with permission.^[^ ^62^ ^]^ Copyright 2016, Nature Publishing Group. f) Schematic of an all‐ceramic SSA consisting of a TiN IR reflector, a TiNO absorbing layer, and ZrO_2_ and SiO_2_ ARCs. g) Absorptance spectra of the all‐ceramic SSA at different fabrication stages. h) Absorptance spectrum of the all‐ceramic SSA over the entire UV–vis–IR range, AM 1.5G solar spectrum, and a 727 °C blackbody radiation spectrum. (f–h) Reproduced with permission.^[^ ^66^ ^]^ Copyright 2019, Elsevier.

Multilayers of alternating metal and dielectric (ceramic) thin films have been intensively explored as SSAs since they have a simple configuration and a facile fabrication process (**Figure** **9**a). The high and selective absorption of multilayer absorbers results from the interference effects in optical resonators and the high optical losses of some metals, such as W, Cr, Ti, and Mo.^[^ ^62^, ^102^, ^103^, ^104^, ^105^, ^106^, ^107^, ^108^ ^]^ Wang et al. fabricated a high‐performance multilayer SSA of W/SiO_2_/W/Si_3_N_4_/SiO_2_ with a considerably high $\bar{\alpha }$ of 95% and long‐term thermal stability at 600 °C in ambient conditions (Figure 9b).^[^ ^102^ ^]^ By combining the plasmon resonance with cavity resonance, Wu et al. numerically demonstrated an SSA by placing W nanospheres on multilayer W–SiO_2_ thin nanofilms, achieving an ultrahigh $\bar{\alpha }$ of 95% and a low $\bar{\varepsilon }$ of 5% at 100 °C simultaneously.^[^ ^103^ ^]^ Multilayered SSAs of Ti‐AlN with various colors (black, purple, green, red, and orange) were even developed. Among them, the purple SSA showed the best optical performance: a $\bar{\alpha }$ of 94% and a low $\bar{\varepsilon }$ of 5% (Figure 9c).^[^ ^107^ ^]^ Extremely stable W–HfO_2_ and Mo–HfO_2_ based multilayer SSAs were presented to offer excellent stability at extremely high temperatures of 1000–1100 °C in a vacuum (Figure 9e).^[^ ^62^, ^105^ ^]^ It should be noted that those periodic metal–dielectric multilayers with fixed metal (and dielectric) thicknesses are also called 1D PhCs.

Multilayer thin films of transition metal nitrides and oxy‐nitrides deposited on metallic reflectors are also promising SSAs. Titanium oxy‐nitride (TiNOX)‐based SSAs is one the most widely used commercial SSAs with an $\bar{\alpha }$ of ≈95% and an $\bar{\varepsilon }$ of ≈5% at 100 °C.^[^ ^109^ ^]^ Other metal nitrides and oxy‐nitrides, such as CrAlON, WAlON, NbAlON, TiAlN, TiSiN, and TiAlSiN, have also been reported as intrinsic materials for SSAs.^[^ ^110^, ^111^, ^112^, ^113^, ^114^ ^]^

#### 4.2.2 PhCs

**Figure 10:**
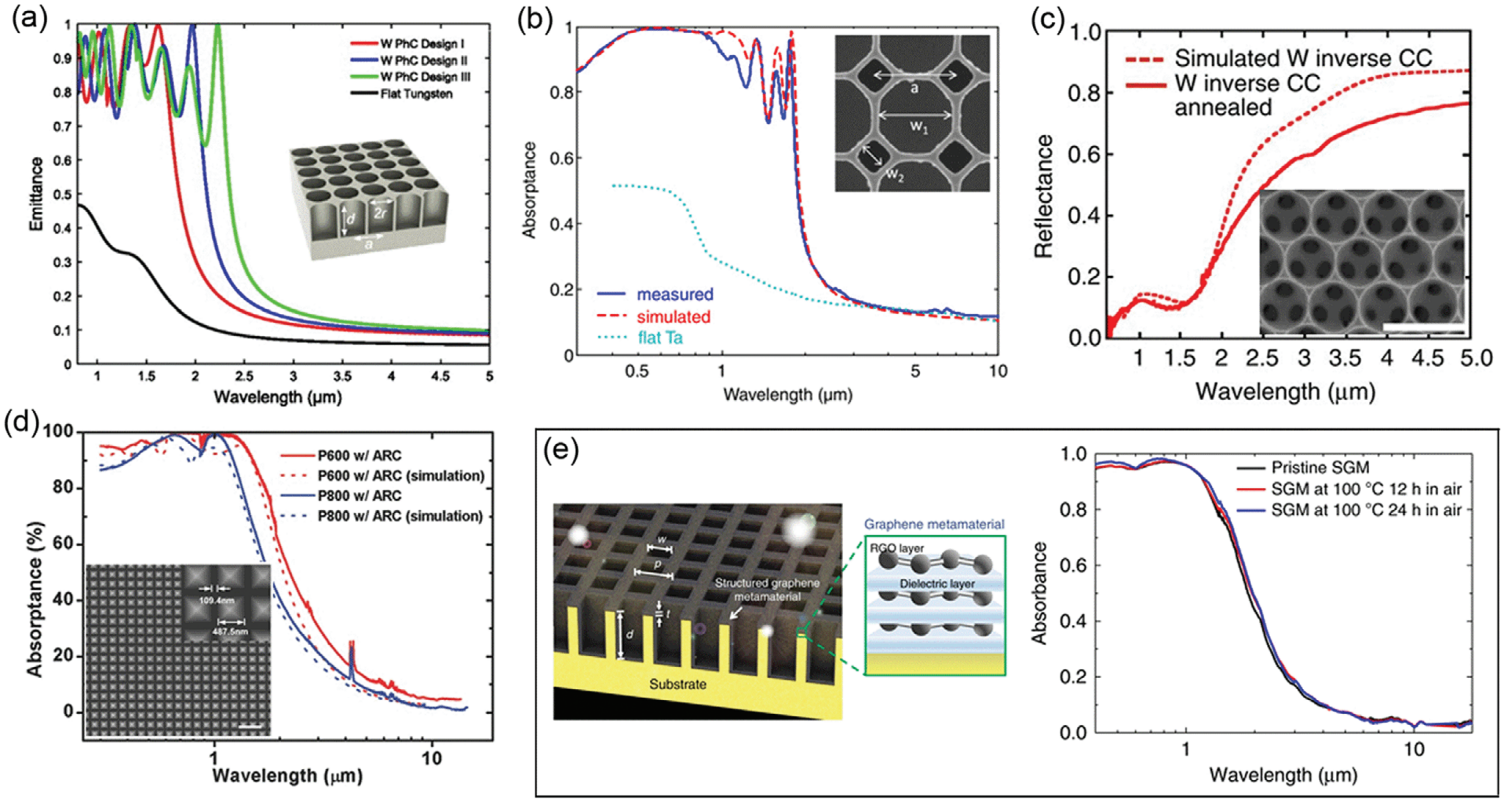
a) Schematic and absorptance/emissivity spectra of 2D W photonic crystals (PhCs). Reproduced with permission.^[^ ^73^ ^]^ Copyright 2012, National Academy of Sciences. b) SEM image and absorptance spectra of a Ta PhCs fabricated by nanoimprinting lithography. Reproduced with permission.^[^ ^115^ ^]^ Copyright 2015, Optical Society of America. c) SEM image and reflectance spectra of a 3D W PhC. Scale bar, 1 µm. Reproduced with permission.^[^ ^118^ ^]^ Copyright 2013, Nature Publishing Group. d) SEM image and absorptance spectra of a 3D Ni nanopyramids PhC. Scale bar, 3 µm. Reproduced with permission.^[^ ^119^ ^]^ Copyright 2015, Wiley‐VCH. e,f) Schematic and absorptance spectra of a 2D graphene–Cu PhC. Reproduced with permission.^[^ ^120^ ^]^ Copyright 2020, Nature Publishing Group.

PhCs are periodic optical nanocavities that can interact with the light (photons) in the same way that crystal lattices interact with electrons. For sunlight harvesting, 1D, 2D, and 3D metallic PhCs were demonstrated to pursue highly selective and broadband solar absorption.^[^ ^54^ ^]^ Differing from other kinds of SSAs with nanoscale thickness, 2D and 3D PhCs were microscale‐thick cavities made from bulky metals by lithographic methods.^[^ ^73^, ^115^, ^116^ ^]^ As a result, both the robust structure and large thickness endow PhCs with exceptional thermal stability up to 1400 °C. In 2012, Yeng et al. fabricated a 2D W PhC as a solar absorber in high‐temperature solar‐thermal systems by interference lithography, exhibiting high solar absorption of around 90% (**Figure** **10**a).^[^ ^73^ ^]^ A 2D PhC based on tantalum (Ta) cavities was reported with a $\bar{\alpha }$ of 92% and a low ${\bar{\varepsilon }}_{100{\;}^{\circ }\text{C}}$ of 12%, which was fabricated by the nanoimprint lithography method (Figure 10b).^[^ ^115^ ^]^ A Ru–HfO_2_–Al_2_O_3_ metallic dielectric PhCs can even sustain its solar selective absorption at temperatures as high as 1000 °C for 24 h in a 95% Ar and 5% H_2_ condition.^[^ ^117^ ^]^ Moreover, a 3D W PhC with an HfO_2_ coating fabricated using self‐assembled colloidal crystals as the template was even thermally stable at 1400 °C in Ar (Figure 10c).^[^ ^118^ ^]^ Li et al. experimentally developed a 3D Ni PhC of nanopyramids with a 95% solar absorptance and 10% thermal emissivity, which was one of the best‐performance PhCs and maintained its performance at 800 °C in a vacuum (Figure 10d).^[^ ^119^ ^]^ More recently, graphene was adopted into Cu PhCs as a solar absorbing medium, and achieved an $\bar{\alpha }$ of 92% and an ultralow $\bar{\varepsilon }$ of 4%, leading to high solar‐thermal efficiency of 88.1% at 100 °C under 1 sun (Figure 10e).^[^ ^120^ ^]^

### 4.3 Plasmon Resonance‐Enhanced SSAs

The excitation of resonance endows the structures with the exceptional capability of strong light–matter interaction and light confinement. For instance, when the metallic nanoparticles interact with electromagnetic waves (light), electric polarization of the surface free electrons is caused by the electric component of light, resulting in localized surface plasmon resonance (LSPR).^[^ ^121^ ^]^ As a result, at resonance, metallic nanoparticles such as Au, Ag, and Al show enhanced absorption cross‐sections that are larger than their geometric cross‐sections. SSAs made of metal nanoparticles and ceramic matrixes, known as cermet, exactly exploit the LSPR‐enhanced absorption of metal nanoparticles.^[^ ^122^ ^]^ Furthermore, magnetic resonance can be induced by placing metal nanoparticles on a metallic film separated by a dielectric spacer.^[^ ^123^, ^124^, ^125^, ^126^ ^]^ In this case, the resulting plasmonic structure can provide both electric and magnetic response to the incident light, resulting in near‐perfect absorption. PMAs that consist of an array of lithography patterned metal nanoparticles are built based on electric and magnetic resonances.

#### 4.3.1 Cermet SSAs

**Figure 11:**
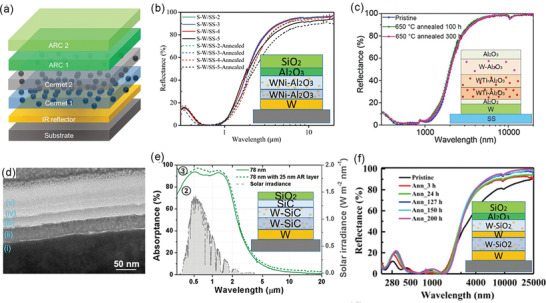
a) Schematic of graded cermet‐based SSAs composed of an IR reflector, two or more layers of graded cermets, and two or more layers of ARCs. b) Reflectance spectra of WNi–Al_2_O_3_ cermet‐based SSAs. Reproduced with permission.^[^ ^129^ ^]^ Copyright 2015, Wiley‐VCH. c) Reflectance spectra of WTi–Al_2_O_3_ cermet‐based SSAs. Reproduced with permission.^[^ ^134^ ^]^ Copyright 2020, Elsevier. d,e) TEM image and absorptance spectra of W–SiC cermet‐based SSAs. Reproduced with permission.^[^ ^131^ ^]^ Copyright 2020, Wiley‐VCH. f) Reflectance spectra of SSAs based on W–SiO_2_ cermets and W multilayers. Reproduced with permission.^[^ ^132^ ^]^ Copyright 2019, Elsevier.

Cermet SSAs have attracted intensive interests due to their great spectral selectivity. High‐performance cermet SSAs generally have two or more layers of cermet with gradient metal fraction that are fabricated by co‐sputtering methods, accompanied by a bottom IR reflector and top antireflection layers (**Figure** **11**a).^[^ ^30^, ^127^, ^128^ ^]^ The gradient metal fraction leads to the gradient refractive index from the bottom to top, and therefore minimized surface reflection. W is the most frequently used metal in cermets because of its high optical loss and high‐temperature reliability.^[^ ^128^, ^129^, ^130^, ^131^, ^132^, ^133^, ^134^, ^135^ ^]^ In 2015, Cao et al. demonstrated a multilayer cermet of W and Ni nanoparticles and a Al_2_O_3_ host by a co‐sputtering method, showing a high $\bar{\alpha }$ of 90% and a low $\bar{\varepsilon }$ of 15% at 500 °C, as well as thermal stability at 600 °C (Figure 11b).^[^ ^129^ ^]^ Later on, a WTi–Al_2_O_3_ cermet‐based SSA with better spectral selectivity ($\bar{\alpha }$ of 92% and $\bar{\varepsilon }$ of 10% at 500 °C) and comparable thermal stability was reported.^[^ ^130^ ^]^ Furthermore, by the use of more stable α‐phase W as the IR reflector, the $\bar{\varepsilon }$ at 500 °C of WTi–Al_2_O_3_ was reduced to 8%, and the tolerable temperature was increased to 650 °C (Figure 11c).^[^ ^134^ ^]^ By combining the W‐based cermets and metal–dielectric multilayer films, high‐performance and thermally stable (600 °C) SSAs were fabricated to provide an extremely high $\bar{\alpha }$ of 96% and an ultralow $\bar{\varepsilon }$of 4% at 82 °C (Figure 11f).^[^ ^132^, ^133^ ^]^ More recently, Raza et al. developed a W–SiC based cermet absorber to simultaneously offer a high $\bar{\alpha }$ of 95% and a low $\bar{\varepsilon }$ of 5% at 100 °C, which was thermally stable at 777 °C in a vacuum (Figure 11d,e).^[^ ^131^ ^]^ In addition to W, other refractory and lossy metals including Mo, Ta, Cr, Ni, and TiN were also exploited to construct cermets.^[^ ^136^, ^137^, ^138^ ^]^ The main concern of cermet SSAs is their instability at extremely high temperatures mainly caused by two factors. The sputtering metal nanoparticles embedded in ceramic oxides are quite small in size (≈10 nm), which are easy to be damaged by diffusion and oxidation. Besides, delamination cracks are found in some high‐performance cermet SSAs that made of at least five‐layer films.

#### 4.3.2 PMAs

**Figure 12:**
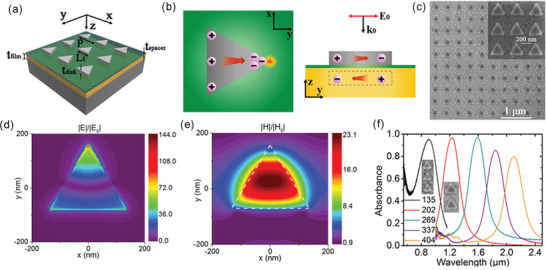
a) Schematic of a plasmonic metamaterial absorber (PMA) consisting of a periodic array of triangular Ag disks, an Al_2_O_3_ spacer, and an Au ground layer. b) Absorption mechanism of the PMA based on out‐of‐plane plasmonic coupling and lightning rod effect, which provide both strong electric and magnetic field enhancements. c) SEM image of the PMA. d) Electric field enhancement and e) magnetic field enhancement in the PMA. f) Absorption spectra of the PMAs with variable nanodisk sizes. Reproduced with permission.^[^ ^125^ ^]^ Copyright 2017, American Chemical Society.

PMAs are another kind of SSA enabled by the plasmonic resonance of nanoparticles. When the size of metal nanoparticles exceeds the magnetic skin depth (20–50 nm for metals), not only electric polarization but also magnetic polarization is induced in nanoparticles by the incident light.^[^ ^139^ ^]^ By disposing such metal nanoparticles on a continuous metal film with a dielectric spacer, the antiparallel currents induced in the metallic nanoparticles and the metallic film will form an enhanced magnetic field. With this special design, the PMAs can generate both electric and magnetic responses to the incident light.^[^ ^123^, ^124^ ^]^ In most previously reported PMAs, metal nanoparticles with a size of hundreds of nanometers are lithographically patterned into a periodic array.^[^ ^74^, ^123^, ^124^, ^125^, ^140^, ^141^, ^142^, ^143^ ^]^ The absorption properties of PMAs strongly depend on the nanoparticle size, the spacer thickness, and the nanoparticle shape. In 2017, by the utilization of triangular nanodisks, Li et al. proposed Ag/Al_2_O_3_/Au PMAs with both near‐perfect (>95%) absorption and large local field enhancement at their resonant wavelengths (**Figure** **12**).^[^ ^125^ ^]^ Wan et al. reported an Au/amorphous C/Au selective metasurface absorber with high 90% absorption in the wavelength range of 0.4–1.0 µm and reduced absorption of only 20% at 1.6 µm.^[^ ^143^ ^]^

**Figure 13:**
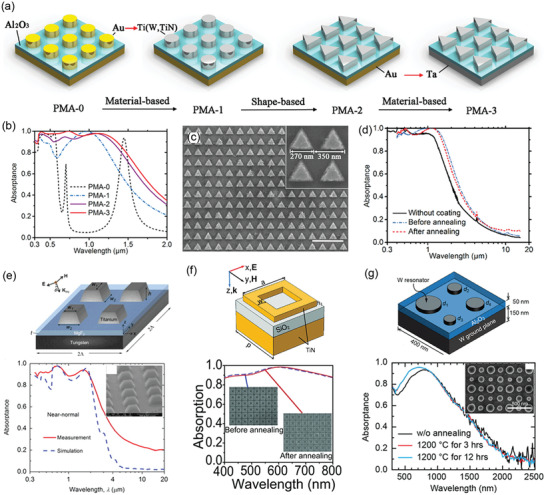
a) Schematic of hybrid‐strategy (structure, material, and shape‐based) plasmonic metamaterial absorbers (PMAs) with triangular nanodisks. b) Absorptance spectra of PMAs in (a). c) SEM image and d) absorptance spectra of PMA‐3 before and after annealing at 727 °C in argon. Scale bar, 1 µm. (a–d) Reproduced with permission.^[^ ^74^ ^]^ Copyright 2018, Wiley‐VCH. e) Schematic, SEM image, and absorptance spectra of a Ti/MaF_2_/W PMA. Reproduced with permission.^[^ ^144^ ^]^ Copyright 2015, Elsevier. f) Schematic, SEM image, and absorptance spectra of a TiN/SiO_2_/TiN PMA with ring‐like nanodisks. Reproduced with permission.^[^ ^141^ ^]^ Copyright 2014, Wiley‐VCH. g) Schematic, SEM image, and absorptance spectra of a W/Al_2_O_3_/W PMA with multisized nanodisks. Reproduced with permission.^[^ ^140^ ^]^ Copyright 2018, American Chemical Society.

To capture the full‐spectrum sunlight, nanoparticles of metals with high optical losses, like W, Mo, Ti, Ni, and Ta are more attractive in solar thermal energy harvesting due to their stronger absorption and refractory properties. As shown in **Figure** **13**, Li et al. proposed a 240‐nm‐thick hybrid‐strategy PMA made of triangular Ti nanodisks/Al_2_O_3_/Ta, showing both a high $\bar{\alpha }$ of 91% and a low $\bar{\varepsilon }$ of 7% at 100 °C (24% at 727 °C). Strong and selective sunlight absorption is realized by adopting a hybrid of structure‐based, material‐based, and shape‐based strategies in the absorber design. Coated with a surface layer of Al_2_O_3_, the PMA is thermally stable up to 727 °C.^[^ ^74^ ^]^ Wang et al. reported a Ti/MgF_2_/W PMA that showed >90% absorption for most of the solar wavelengths and reduced absorption in the IR region ($\bar{\varepsilon }$ of 20% at 100 °C, Figure 13e).^[^ ^144^ ^]^ Recently, a W/Al_2_O_3_/W PMA comprising four W‐nanodisks of different sizes in a unit cell was fabricated to broaden the absorption band in the visible‐NIR region, leading to an $\bar{\alpha }$ of 83% and a thermal emissivity $\bar{\varepsilon }$ < 10%. The PMA with an extremely stable HfO_2_ coating can sustain its performance at temperatures up to 1200 °C in a vacuum (Figure 13g).^[^ ^140^ ^]^ Titanium nitride (TiN) was also used as resonant nanoparticles in the development of PMAs due to its tunable optical properties and high‐temperature stability up to 800 °C (Figure 13f).^[^ ^141^ ^]^ Compared to cermets, PMAs can offer comparable spectral selectivity and better high‐temperature stability due to the larger nanoparticles and the reduced number of layers. The main challenges in PMAs are the expensive nanopatterning processes such as e‐beam lithography, limiting their large‐scale fabrication. It was demonstrated that the gap size and orientation of nanodisks in PMAs only have limited influence on their optical performance.^[^ ^74^ ^]^ By using low‐cost techniques such as short‐term sputtering,^[^ ^145^, ^146^ ^]^ nanoimprint lithography,^[^ ^115^ ^]^ and solution‐based processes,^[^ ^126^ ^]^ the mass production of PMAs at low costs would be realized.

**Table** **1** compares different classes of SSAs regarding their structural complexity, fabrication process, cost, spectral selectivity, thermal stability, and potential for water production. It should be noted here that each property given in Table 1 belongs to a typical, high‐performance SSA of different classes. For instance, the thermal stability of cermet‐based SSAs is evaluated as “++,” while that of PhCs is “+++,” which indicates that most of the PhCs show better thermal stability than cermets, and there may be some exceptions.

**Table 1:** Comparison of the different classes of SSAs

Classes		Structure complexity	Fabrication process	Cost	Spectral selectivity	Thermal stability	Water production
Intrinsic SSAs		+^a)^	PVD, CVD	+	+	+	+
Optical cavity	Multilayer thin films	++	PVD, CVD	++	++	+	++
	PhCs	+++	Lithography	+++	++	+++	+++
Plasmon resonance	Cermet	++	PVD, CVD	++	++	+	++
	PMAs	+++	Lithography	++++	+++	++	++

^a)^ The number of “+” indicates the degree of the complexity, cost, spectral selectivity, thermal stability, and potential for water production.

### 4.4 All‐Ceramic SSAs

**Figure 14:**
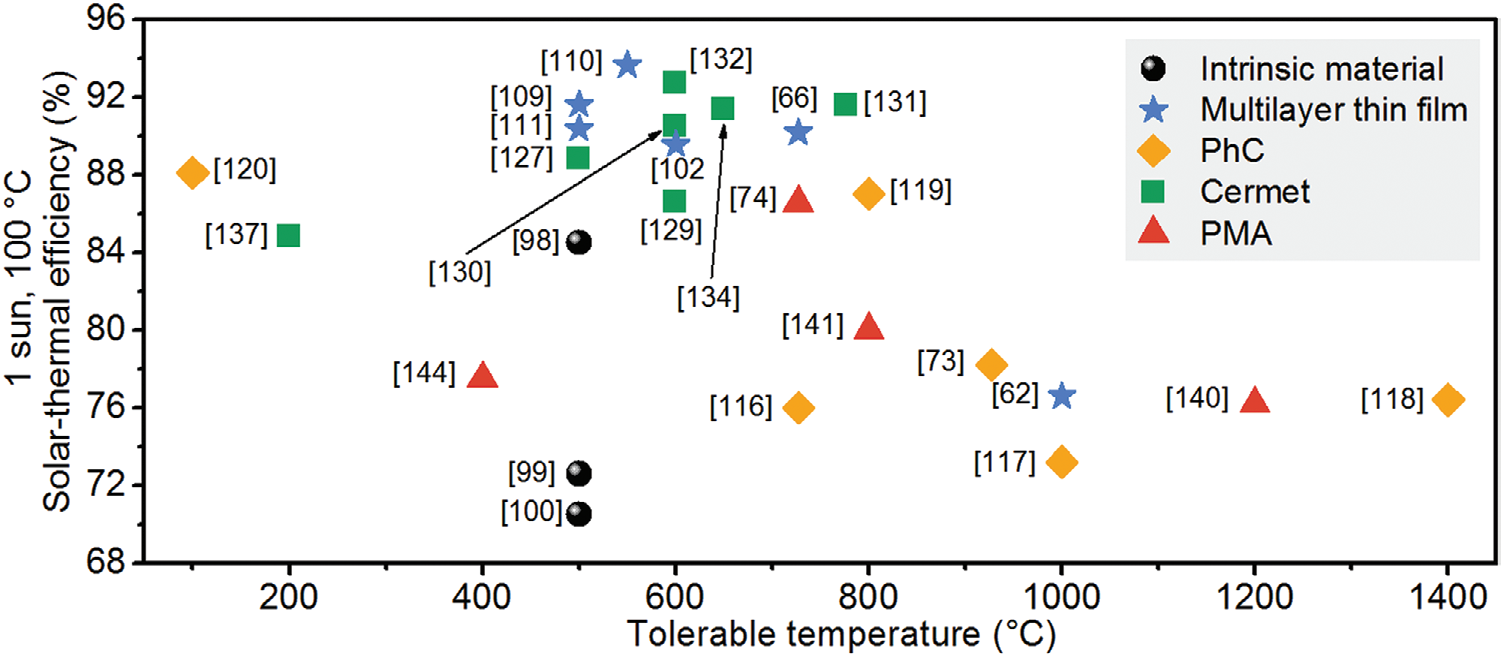
Comparison of tolerable temperature and solar‐thermal conversion efficiency (under 1 sun and 100 °C) for recently reported SSAs using different strategies. Solar‐thermal efficiency values are calculated from the absorptance spectra in cited references.

**Figure** **14** summarizes the overall performance of the recently reported SSAs mentioned above. Compared to SSAs made of intrinsically selective materials, those enabled by optical cavities and plasmon resonances show more competitive overall performance, especially in spectral selectivity and thermal stability. Plasmon‐resonance SSAs can provide strong and highly selective solar absorption, but their maximum tolerable temperatures are limited to around 700 °C so far due to the instability of metal nanoparticles. Optical cavity resonators that are more thermally stable usually suffer from insufficient solar absorption and spectral selectivity, as well as concerns on their mass production. For almost all the reported SSAs, metals are indispensable components acting as IR reflectors and absorbing media, but the usage of metals also brings a lot of issues.^[^ ^66^ ^]^ For high‐temperature applications, refractory metals such as W, Mo, and Ta are employed as building blocks, but they typically exhibit relatively larger IR emission (>5% at 100 °C) than Au and Ag, reducing the spectral selectivity. Moreover, metallic nanoparticles and thin films are not thermally stable due to the oxidation, diffusion, and detachment in the metal‐dielectric interfaces. Recently, Li et al. proposed to construct metal‐free SSAs using all‐ceramic components that are more robust at high temperatures and have tunable optical properties.^[^ ^66^ ^]^ A proof‐of‐concept all‐ceramic SSA with multilayer TiN/TiNO/ZrO_2_/SiO_2_ films was experimentally demonstrated by facile lithography‐free film deposition methods (Figure 9f). Taking advantage of the excellent IR reflection of TiN and gradient absorption media, the all‐ceramic SSA offered a solar absorptance an $\bar{\alpha }$ of 92% and an ultralow $\bar{\varepsilon }$ of 17% at 727 °C, which was thermally stable at 727 °C for 150 h in argon and vacuum conditions (Figure 9g,h).

## 5 Mechanism and Recent Progress of IRSEs

**Figure 15:**
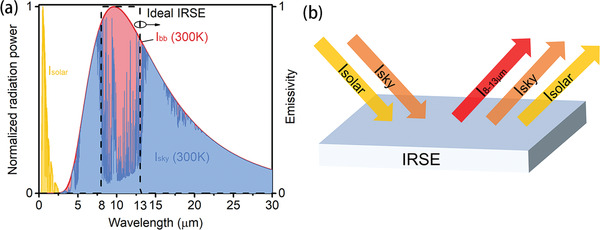
a) Normalized radiation power of solar, sky, and blackbody, as well as the emissivity of ideal IR selective emitter. b) Mechanism of IR selective emitter (IRSE).

As discussed in Section 3.2, both the cooling temperature and the cooling power are two main concerns for radiative cooling. Ideal IR emitters should have near‐perfect and highly selective IR emission only in the atmospheric window to achieve both large temperature drop and remarkable cooling power (**Figure** **15**). So here we use two parameters to evaluate the cooling performance of emitters. One is average emissivity over the main atmospheric window, ε_8–13 µm_ (Equation (12)), which illustrates the initial cooling power of an emitter. The other is the selectivity of IR spectrum *η_ε_*, (Equations (13) and (14)), corresponding to radiative cooling efficiency and potential of temperature drops^[^ ^82^, ^147^, ^148^ ^]^

(12) $$\begin{array}{c} \varepsilon_{8-13\;\mu \text{m}}=\frac{\int_{8\;\mu \text{m}}^{13\;\mu \text{m}}\text{d}\lambda I_{\text{BB}}(\lambda ,T_{\text{amb}})\varepsilon (\lambda )}{\int_{8\;\mu \text{m}}^{13\;\mu \text{m}}\text{d}\lambda I_{\text{BB}}(\lambda ,T_{\text{amb}})} \end{array}$$

(13) $$\begin{array}{c} \varepsilon_{0-\infty }=\frac{\int_{0}^{\infty }d\lambda I_{\text{BB}}(\lambda ,T_{\text{amb}})\varepsilon (\lambda )}{\int_{0}^{\infty }d\lambda I_{\text{BB}}(\lambda ,T_{\text{amb}})} \end{array}$$

(14) $$\begin{array}{c} \eta_{\varepsilon }=\frac{\varepsilon_{8-13\;\mu \text{m}}}{\varepsilon_{0-\infty }} \end{array}$$

According to the above‐mentioned discussion in this section, *η_ε_* should be as high as possible to maximize its cooling temperature, while ε_8–13 µm_ should be optimized for a remarkable cooling power. In the following section, emitters being introduced will be evaluated by these two parameters ε_8–13 µm_ and *η_ε_*_,_ at *T*_amb_ = 300 K.

As we mentioned above, the strategies used to achieve high emissivity in the IR region are similar to those in the solar spectral region. Therefore, the IRSEs can also be classified into three classes: intrinsic IRSEs, photonic structures‐enabled IRSEs, and particle resonance‐enabled IRSEs. Specifically, IRSEs based on 1D photonic structures are those multilayer thin films of two alternating materials (such as HfO_2_ and SiO_2_), also known as 1D photonic crystals. IRSEs with particle resonances include metamaterial absorbers made of metal/dielectric/metal, and particle dispersed composites. Metamaterial absorbers operating in the mid‐IR region share the same structure with those operating in the visible wavelengths but have larger feature sizes.

### 5.1 Intrinsic IRSEs

**Figure 16:**
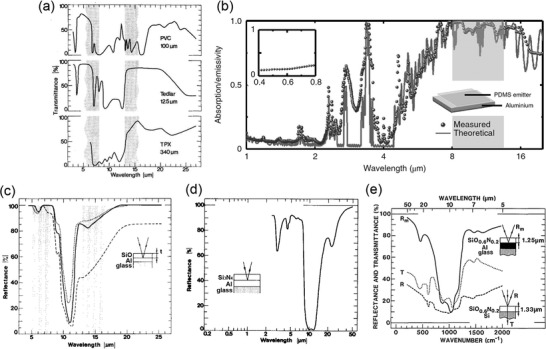
Spectra of intrinsic emitters with materials of a) PVC, Tedlar, TPX. Reproduced with permission.^[^ ^147^ ^]^ Copyright 1981, AIP Publishing. b) PDMS. Reproduced with permission.^[^ ^50^ ^]^ Copyright 2019, Nature Publishing Group. c,d) SiO and Si_3_N_4_. Reproduced with permission.^[^ ^82^ ^]^ Copyright 1982, Elsevier. e) SiO*_x_*N*_y_*. Reproduced with permission.^[^ ^151^ ^]^ Copyright 1983, Optical Society of America.

Some materials have strong absorption bands within 8–13 µm while weaker out of this region. Controlling the thickness of such materials can generate high emission only in the atmospheric window. Polymers have absorption bands in the IR region and its absorption wavelengths (wavenumbers) depend on the type of polymers. Those absorptions are caused by band vibrations when IR interacts with the chemical bonds. Masses of atoms, the strength of chemical bonds, and the structures of molecules all affect the final absorption spectrum.^[^ ^149^ ^]^ Thus, polymers can be identified by the IR spectrum, and can also be selected for selective IR emission (absorption). A single layer of polyvinylchloride (PVC), polyvinylfluoride (Tedlar), and poly 4 methylpentene (TPX) were reported to generate strong emission in 8–13 µm and high transmittance elsewhere (**Figure** **16**a).^[^ ^147^ ^]^ Backed with a reflective layer, IRSE can be prepared. Also, Zhou et al. and Czapla et al. coated a single polydimethylsiloxane (PDMS) thin film with a controlled thickness on Al to get another well‐performed polymer‐based IRSE (Figure 16b).^[^ ^50^, ^150^ ^]^ Those works proved the possibility of choosing a proper polymer with its intrinsic absorption for IRSE in the atmospheric window.

Apart from polymer, some ceramic materials also exhibit intrinsically selective IR emission. Silicon‐based ceramics, including SiO, Si_3_N_4_, and SiO*_x_*N*_y_*, were used as a single emission layer because the absorption bands of the Si—O bond and Si—N bond are within 8–13 µm. Granqvist et al. deposited SiO and Si_3_N_4_ on top of the Al film to realize great IRSEs (Figure 16c,d).^[^ ^82^ ^]^ Later, SiO*_x_*N*_y_* was also explored to achieve even higher emission (Figure 16e).^[^ ^151^, ^152^ ^]^ Different from polymers, ceramic materials do not have the aging problem and show better durability under solar (containing UV) exposure. However, the above ceramics were fabricated by a vacuum process with a relatively higher cost than polymers. Easier methods can be considered to make ceramic IRSEs potential for large‐scale application in the future.

Generally, single layers with selective intrinsic emission can realize IRSEs with an extremely simple structure. However, the wavelength flexibility is limited and the emission value in the atmospheric window will also be suppressed by surface reflection caused by the Reststrahlen band, so the emission cannot be further improved once it reaches a certain saturation level.

### 5.2 Multilayer Thin Films‐Enabled IRSEs (1D Photonic Structures)

Since the spectral selectivity of single‐layer intrinsic materials is limited, some researchers used multilayer thin films to generate a desired IRSE. Such a structure is based on interference effects that the wave reflected by the upper boundary of a thin film interferes with the wave reflected by the lower boundary, leading to a coupling phenomenon with either reflection enhancement or reflection cancellation. One active interference can control the spectrum in a certain narrow wavelength with a specific optical path of a functional layer. When multilayers are applied with a controlled thickness of each layer, a broadband spectrum can be realized.

**Figure 17:**
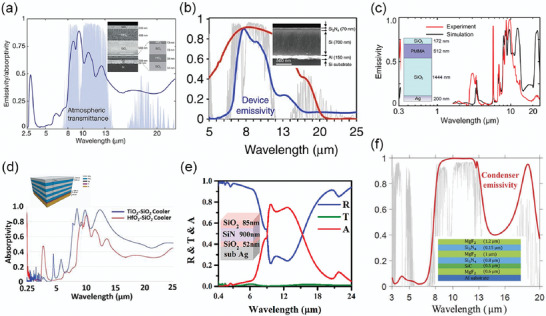
Spectra of interference‐based multi‐layers emitters with materials of a) HfO_2_–SiO_2_. Reproduced with permission.^[^ ^46^ ^]^ Copyright 2014, Nature Publishing Group. b) Si_3_N_4_–Si. Reproduced with permission.^[^ ^52^ ^]^ Copyright 2016, Nature Publishing Group. c) SiO_2_–PMMA. Reproduced with permission.^[^ ^153^ ^]^ Copyright 2017, SPIE. d) TiO_2_–SiO_2_. Reproduced with permission.^[^ ^154^ ^]^ Copyright 2020, Elsevier. e) SiO_2_–SiN. Reproduced with permission.^[^ ^155^ ^]^ Copyright 2019, American Chemical Society. f) MgF_2_–Si_3_N_4_–SiC coated on metal film (Ag or Al). Reproduced with permission.^[^ ^84^ ^]^ Copyright 2020, Taylor & Francis.

In 2014, a multilayer structure with seven layers of HfO_2_ and SiO_2_ was reported, based on interference effects, to realize selective thermal emission.^[^ ^46^ ^]^ This IRSE achieved daytime cooling (≈5 °C drop) for the first time, benefited from its excellent IR selectivity, and strong solar reflection (**Figure** **17**a). Later, a simpler IRSE was proposed with a three‐layers structure on Si substrate in 2016 (Figure 17b).^[^ ^52^ ^]^ Working inside a vacuum chamber under sealing of a ZnSe window, this IRSE obtained maximum and average temperature reduction of 42 and 37 °C, respectively, in a 24 h day–night cycle. Its excellent IR selective emission and thermal insulation contributed to such substantial cooling temperatures. Subsequently, SiO_2_–PMMA,^[^ ^153^ ^]^ TiO_2_–SiO_2_,^[^ ^154^ ^]^ SiO_2_–SiN,^[^ ^155^ ^]^ MgF_2_–Si_3_N_4_–SiC^[^ ^84^ ^]^ based multilayer structure were also reported to generate IRSEs (Figure 17c–f).

Compared to single‐layer structures, multilayer structures have higher degrees of freedom in controlling spectrum shape and emission intensity. However, emission spectra of those 1D photonic structures are sensitive to thicknesses of each layer and require accuracy of nanolevel, thus the fabrication process is more complicated and challenging at a higher cost than single‐layer thin films.

### 5.3 Particle Resonance‐Enabled IRSEs

#### 5.3.1 Metamaterial Absorbers

In order to further improve IR selectivity and realize near‐perfect IRSEs, some metasurfaces with 2D or 3D structures were also investigated. Two main resonant‐based methods were used including surface plasmonic resonances (SPRs) and surface phonon–polariton resonances (SPs). The SPR stems from collective conduction electron oscillations in metallic nanoparticles,^[^ ^125^, ^156^ ^]^ while the SPs result from lattice vibrations in polar dielectric particles.^[^ ^157^, ^158^ ^]^ Both resonances can be stimulated by incident light and generate strong emission (absorption) at a certain wavelength. SPR is more commonly used for SSAs as the resonance band of metals is usually in the visible and near‐IR region. But still, some researchers shifted those bands into mid or even far‐IR by adjusting the structure scale. Compared to SPR, SPs are widely used for spectrum manipulation in IR or even terahertz wavelengths.

**Figure 18:**
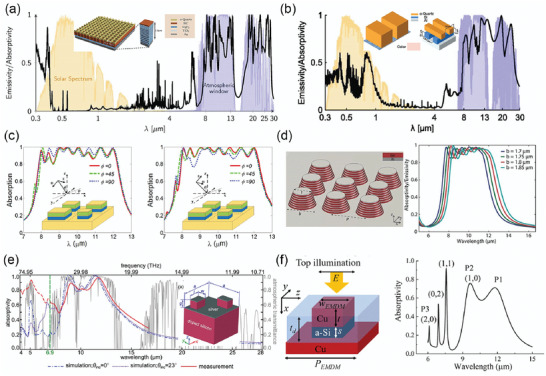
Spectra of emitters enhanced by pattern‐resonance through a) quartz‐SiC 2D periodic structure. Reproduced with permission.^[^ ^53^ ^]^ Copyright 2013, American Chemical Society. b) Quartz‐Si 3D periodic structure. Reproduced with permission.^[^ ^89^ ^]^ Copyright 2013, AIP publishing. c) Parallel LC circuit model. Reproduced with permission.^[^ ^75^ ^]^ Copyright 2014, Optical Society of America. d) Ge–Si CMM pillars. Reproduced with permission.^[^ ^159^ ^]^ Copyright 2015, Wiley‐VCH. e) Metal‐loaded dielectric resonators. Reproduced with permission.^[^ ^161^ ^]^ Copyright 2017, Wiley‐VCH. f) 3D periodic cubic EMDM resonators. Reproduced with permission.^[^ ^160^ ^]^ Copyright 2017, Optical Society of America.

In 2013, Fan's group at Stanford University proposed two SPs‐based structures to realize IRSE with strong emission in two atmospheric windows (8–13 and 18–30 µm) and low emission elsewhere (**Figure** **18**a,b).^[^ ^53^, ^89^ ^]^ Quartz (SiO_2_) was used in both two design since it has a significant resonance at 9.3 µm. SiC was also adopted into one structure with a resonance peak at 12.5 µm. However, the second window only exists in dry climates and such selectivity leads to additional heat absorption from the atmosphere and suppresses temperature drop, especially in humid regions. Later, a parallel LC circuit model was reported in 2014 to generate strong emission only focusing on 8–13 µm (>0.9), through multibands plasmonic resonances (Figure 18c).^[^ ^75^ ^]^ But this work only showed simulation results and no experimental samples were demonstrated. In 2015, a metamaterial of conical metal–dielectric pillars with alternating layers of Al and Ge was proposed to realize an excellent IRSE, not only in theory but also in the experiment (Figure 18d).^[^ ^159^ ^]^ This metamaterial was claimed to possess a cooling power of 116.6 W m^−2^.

Some different designs based on surface resonance (SPR or SPs) were also tried to construct IRSE (Figure 18e,f) and superior performances can be observed in the IR region.^[^ ^160^, ^161^, ^162^ ^]^ However, the surface patterns of those metamaterial emitters require a high‐accuracy fabrication process, which restricts their large‐scale application. Also, it is still challenging to minimize the solar absorption for those metamaterial emitters as some additional resonance cannot be neglected in the visible or near‐IR region. To maintain its cooling function in daytime, solar shading structures should be considered.

#### 5.3.2 Particle Dispersed Composites

Apart from spectrum optimization, a simple structure with low‐cost fabrication is becoming new attention, which is important to fulfill real application. Different from the surface patterning process, particles can be prefabricated and its enclosed surface can also generate surface phonon polariton resonance (SPs). Despite its spectrum manipulation is not as flexible as metamaterial emitters, changing materials, size, and concentration of particles provide convenient methods for spectral control.

**Figure 19:**
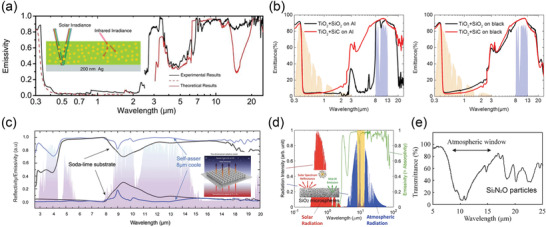
Spectra of emitters enhanced by particle‐resonance through a) SiO_2_–TPX hybrid layer. Reproduced with permission.^[^ ^47^ ^]^ Copyright 2017, AAAS. b) TiO_2_–SiO_2_/SiC double‐layer. Reproduced with permission.^[^ ^164^ ^]^ Copyright 2017, Elsevier. c) SiO_2_ microspheres close‐packed monolayer. Reproduced with permission.^[^ ^163^ ^]^ Copyright 2019, Wiley‐VCH. d) SiO_2_ microspheres paint. Reproduced with permission.^[^ ^165^ ^]^ Copyright 2018, American Chemical Society. e) Si_2_N_2_O particles. Reproduced with permission.^[^ ^166^ ^]^ Copyright 2016, The Ceramic Society of Japan.

SiO_2_ particles are frequently used for the particle‐based IRSEs, as they have a Reststrahlen band in 8–13 µm, which can induce strong absorption (emission) through the SPs effect. In 2017, mixing SiO_2_ spheres with a size of 8 µm into a 50 µm polymer, Zhai et al. fabricated a meter‐scale film, exhibiting emissivity larger than 0.93 over the atmospheric window (**Figure** **19**a).^[^ ^47^ ^]^ Backed with a silver coating, 97% solar reflection and cooling power of 93 W m^−2^ were realized at noontime. Although its IR selectivity is not as close‐ideal as the spectrum generated by metamaterial emitters, it was the first time that a large‐size emitter with excellent daytime cooling was fabricated with a low‐cost method. Different from dispersing SiO_2_ microspheres in a polymer film, the same size of SiO_2_ microspheres were close‐packed as a monolayer on top substrate.^[^ ^163^ ^]^ The diffraction effect of such a periodic lattice not only further enhanced the total emission but also introduced near field emission to far‐field performance. An average thermal emission of 0.98 was achieved over the atmospheric window, but the spectrum was still in broadband emission (Figure 19c). In another work, double layers of nanoparticles were coated separately: SiO_2_ particles were coated at the bottom, functioning as a selective emission layer, while TiO_2_ particles were coated on top to reflect solar energy (Figure 19b).^[^ ^164^ ^]^ This structure realized 0.9 thermal emissions in 8–13 µm and 90% solar reflection. β‐SiC nanoparticles, with Reststrahlen band also in 8–13 µm, was also tried in this work to replace SiO_2_. The broader emission spectrum was shown since commercial β‐SiC has a broader absorption band (Figure 19b). Besides, SiO_2_ microspheres were also painted directly on the substrate to provide strong IR emission (0.94) as well as high solar reflection (Figure 19d).^[^ ^165^ ^]^ It was reported in another work that SiO_2_ particles were heated with Si powders, resulting in Si_2_N_2_O particles for another particle‐based IRSE (Figure 19e).^[^ ^166^ ^]^

Basically, coating particles with SPs effect in the atmospheric window is a simple but effective way to fabricate an IRSE in low‐cost processing. However, no single kind of particles can stimulate high emission exactly in the sky window, which indicates that strong emission in targeted wavelengths may also lead to some parasitic emission in other regions. Therefore, comprehensive structures adopting different aforementioned strategies may be applied together to realize a near‐perfect spectrum and maintain a straightforward fabrication process at the same time.

### 5.4 IR Emission Performance of Reported Emitters

**Figure 20:**
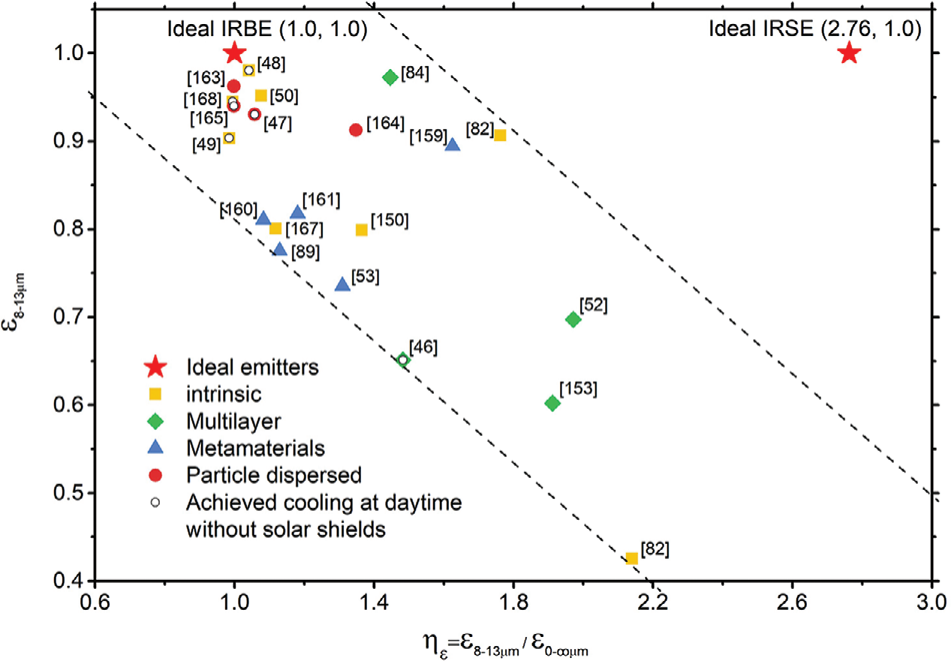
The emissivity over the main atmospheric window (ε_8–13µm_) and the IR selectivity (*η_ε_* = ε_8–13µm_/ε_0‐∞µm_), corresponding to radiative cooling efficiency, of reported emitters. The data were calculated through posted figures and the wavelength ranges were consistent with reported ranges.

Four strategies, including intrinsic IRSEs, multilayer thin films, metamaterial emitters, and particle dispersed composites were reported to fabricate thermal emitters for radiative cooling. Two main parameters describing the cooling performance (ε_8–13 µm_ and *η_ε_*) were calculated for different emitters, based on their published spectrum and plotted in **Figure** **20**.^[^ ^46^, ^47^, ^48^, ^49^, ^50^, ^52^, ^53^, ^82^, ^84^, ^89^, ^150^, ^153^, ^159^, ^161^, ^162^, ^163^, ^164^, ^165^, ^167^, ^168^ ^]^ Many emitters were reported in the last 5 years and some of them exhibit performance close to ideal IRBE. However, all emitters were located between two dash lines in Figure 20, indicating that it is still a challenge to achieve high emissivity and great IR selectivity simultaneously.

Apart from IR selectivity, daytime cooling is another challenge, which requires strong solar reflection (>95%) of the emitters. Although radiative cooling was begun to be investigated around half a century ago, only until 2014 that a first emitter working at daytime was reported.^[^ ^46^ ^]^ Later, some remarkable works for daytime cooling were published in the following years, while many emitters still need additional solar shields to works by blocking solar radiation. And those emitters working at daytime without solar shields still either have IR selective emission but relatively low emissivity or offer high emissivity but poor IR selectivity. It is quite challenging to integrate strong solar reflection, high thermal emissivity, and great IR selectivity in a single emitter so far, which is required for an IRSE with an excellent cooling performance for days and nights.

### 5.5 Solar Rejection

Selective thermal emission can guarantee the potential of the temperature drop of an emitter during nighttime. However, such cooling performance can only maintain during the daytime if the structure absorbs minimum solar heat. Two main strategies were applied for solar rejection, including bottom solar reflector and top solar shield.

When the emitters are transparent to solar light, a bottom solar reflector can be coated on the backside to generate strong solar reflection. Metals were widely used as the bottom reflective layer as it strongly reflects not only the solar radiation but also longer wavelength, which would not damage the selectivity of thermal emission. Among them, silver is the best reflector with the strongest solar reflectance (≈97–98%), followed by aluminum with an average reflectivity of around 90%.

When the emitters have non‐negligible solar absorption that exceeds the IR emission power, a solar shield should be applied on top of the emitters to block solar irradiation. Such a shield can be an opaque plate shading not directly above the emitter, to avoid blocking the atmospheric window. B Bhatia had demonstrated such a method to achieve a 6 °C subambient temperature.^[^ ^166^ ^]^ A Similar method was also applied in the humid area to maximize the cooling effect in the daytime.^[^ ^154^ ^]^ However, this method is impractical if the desired cooling area is large. Thus, shield with strong solar reflection and high mid‐ and far‐IR transmittance was also investigated, like polyethylene (PE) aerogel. 13 °C cooling was observed at noon when this PE aerogel was applied, as its low thermal conductivity could also reduce the convective and conductive heat loss of the emitter.^[^ ^169^ ^]^

### 5.6 Angular Emission

Emitters usually have strong absorption at both low and high incident angles within 8–13 µm, while the atmospheric transmittance reduces from low incident angles to high incident angles.^[^ ^147^ ^]^ Thus the emission (or absorption) at higher angles suppresses the temperature drops of the emitters. If an emitter can offer not only wavelength selectivity but also angular selectivity (high absorption at low incident angles and low absorption at high incident angles, the cooling performance can be further enhanced.^[^ ^170^, ^171^ ^]^ Besides, heat absorption from surrounding high constructions can also be avoided by such thermal beam‐shaping design.^[^ ^172^ ^]^ Zhou et al. proved it experimentally by applying a directional guiding structure on top of a PDMS‐based emitter and successfully improved the temperature drop from 2.5 to 6 °C when higher buildings were surrounded.^[^ ^50^ ^]^

### 5.7 Evaluation of Different Classes of Emitters

**Table** **2** illustrates the comparison of the emitters from different classes at a more practical level. Both intrinsic and particle dispersed emitters have a simple structure, which can be fabricated by a low‐cost fabrication process, such as wet coating. While multilayer thin films and metamaterial emitters had complex structures and expensive manufacturing methods, like physical vapor deposition (PVD), chemical vapor deposition (CVD), and even lithography, so that the cost is generally high. In terms of the optical performance, intrinsic and particle dispersed emitters can offer high IR emissivities but poor IR selectivity, but it is opposite for multilayer thin films and metamaterial emitters. Thus emitters with both strong IR emission and excellent IR selectivity are still urgently demanded in practical applications. The potential for water production at nighttime strongly relies on their optical performance in the IR region. For daytime application, solar reflection should be considered. Intrinsic emitters are relatively easy to achieve high solar reflectance, followed by multilayer thin films and particle dispersed composites. Metamaterial emitters usually have unavoidable solar absorption because of the high‐order resonances in short wavelengths. Since solar power is much larger than the cooling power, the potential of daytime water production mainly depends on the solar reflection levels. Thus intrinsic emitters have the largest potential, followed by multilayer thin films and particle dispersed composites.

**Table 2:** Comparison of the different classes of IRSEs

Classes	Structure complexity	Fabrication process	Cost	IR emissivity	IR selectivity	Solar reflection	Water production (nighttime)	Water production (daytime)
Intrinsic	+^a)^	Wet coating	+	+ + +	+ +	+ + +	+ + +	+ + +
Multilayer	+ + +	PVD/CVD	+ +	+	+ + + +	+ +	+ + +	+ +
Metamaterial	+ + + +	Lithography	+ + +	+ +	+ + +	+	+ +	+
Particle dispersed	+ +	Wet coating	+	+ + +	+	+ +	+ +	+ +

^a)^ The number of “+” indicates the degree of the complexity, cost, IR emissivity, IR selectivity, solar reflection, and potential of water production at nighttime and daytime.

## 6 Current Applications of SSAs on Solar Steam Generation

SSAs enclosed in vacuum tubes have been widely used in solar thermal panels and parabolic trough collectors (PTCs) to generate high‐temperature water and steam.^[^ ^32^, ^76^ ^]^ By 2018, the worldwide capacity of solar‐thermal panels and PTCs reached around 485 GW, which is larger than that of solar cells. However, in this review, we will mainly focus on the application of SSAs on the new ISSG systems.

### 6.1 Commercial SSAs

**Figure 21:**
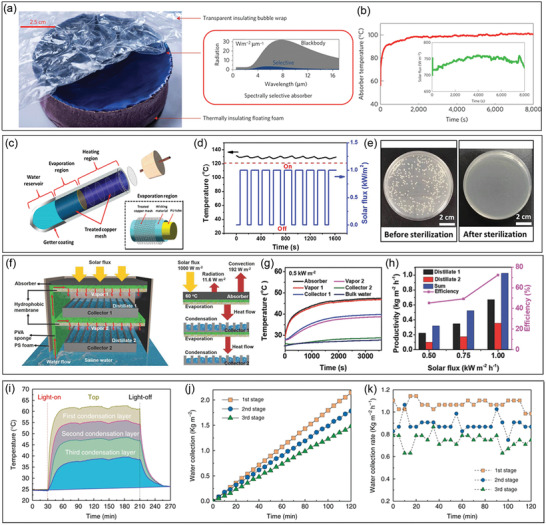
a,b) A ISSG device with a commercial SSA that generates high temperature (>100 °C) steam under 0.75 sun. Reproduced with permission.^[^ ^34^ ^]^ Copyright 2016, Nature Publishing Group. c) Schematic of a portable sterilization device with superheated steam (>121 °C) enabled by a vacuum‐tube sealed SSA. d) Steam temperature under periodically switching‐on and switching‐off solar illumination. e) Photographs of bacteria before and after steam sterilization. c–e) Reproduced with permission.^[^ ^33^ ^]^ Copyright 2019, America Chemical Society. f) Schematic of a two‐stage solar distillation system with a commercial SSA. g) Temperatures of the absorber and steam in stage 1 and 2. h) Water evaporation rates and solar‐to‐vapor efficiency in stages 1 and 2, and the sum. f–h) Reproduced with permission.^[^ ^174^ ^]^ Copyright 2018, Wiley‐VCH. i–k) Steam temperatures and water evaporation rates from different stages in a three‐stage solar distillation system. Reproduced with permission.^[^ ^175^ ^]^ Copyright 2019, Nature Publishing Group.

As shown in **Figure** **21**a, the first demonstration of applying commercial SSAs on steam generation was reported in 2016 by Chen's group.^[^ ^34^ ^]^ An SSA (BlueTec) with a high $\bar{\alpha }$ of 93% and a low IR emissivity ${\bar{\varepsilon }}_{100{\;}^{\circ }\text{C}}$ of 7% was floated on polystyrene foam and covered by polyethylene bubble wrap to generate high‐temperature (>100 °C) steam in ISSG systems under ambient air conditions and unconcentrated solar power (Figure 21b). Water was delivered to the SSA surface by fabric wicks hidden in the polystyrene foam via capillary effects. The smart design significantly suppresses the convective, conductive, and radiative heat losses at the same time. Following the same strategy, Chang et al. reported a 3D porous solar evaporator with a commercial SSA (TiNOX, $\bar{\alpha }$ of 95%, and ${\bar{\varepsilon }}_{100{\;}^{\circ }\text{C}}$ of 5%), and achieved steam >100 °C under 1 sun illumination but a much higher solar‐steam conversion efficiency of 48%.^[^ ^173^ ^]^ The improvement of conversion efficiency is mainly attributed to the amplified effective evaporation area and the change of steam outlet from top to sidewall of the SSA. Furthermore, to generate steam above 121 °C for sterilization under weaker solar flux (<1 sun), they employed a double‐walled solar vacuum tube coated with the commercial SSA (Figure 21c–e).^[^ ^33^ ^]^ Assisted by a hydrophobic evaporator, superheated steam of 100–165 °C is produced under 1 sun with variable evaporator areas. In outdoor measurement, the stable steam temperature is as high as 123 °C under an averaged flux of 0.6 sun. The results of this work demonstrate that by the usage of SSAs, effective outdoor sterilization can be realized in ≈7 h of summer days and ≈5 h of winter days.

Besides the generation of high‐temperature steam, commercial SSAs were also used to enable high‐efficiency freshwater production in multistage distillation devices. By the usage of multistage configuration, the waste heat of the first stage can be utilized by the latter stages, therefore significantly improving the heat utilization efficiency of the system. Xue et al. first reported a two‐stage solar membrane distillation device using a commercial SSA (BlueTec) to capture sunlight (Figure 21f).^[^ ^174^ ^]^ In lab experiments, freshwater production rates of 0.7 and 0.3 kg m^−2^ h^−1^ were achieved in the first and second stages, respectively, leading to a total 1.0 kg m^−2^ h^−1^ and a solar‐thermal efficiency up to 72% under 1 sun illumination (Figure 21g,h). In the outdoor operation, total water evaporation is around 3.67 kg m^−2^ in the daytime under the solar flux ranging from 0.1 to 0.7 sun. Wang et al. demonstrated three‐stage and five‐stage distillation devices with commercial SSAs (BlueTec), achieving water evaporation rates of 2.78 and 3.25 kg m^−2^ h^−1^, respectively (Figure 21i–k).^[^ ^175^ ^]^ Furthermore, Chiavazzo et al. reported a 10‐stage desalination device that also employed the commercial TiNOX SSA and a 3D printed polyethylene film‐based convection reducer.^[^ ^77^ ^]^ A distillation rate as high as 3.0 kg m^−2^ h^−1^ was obtained under 0.9 sun illumination, which is twice larger than most of the state‐of‐the‐art solar desalination systems. SSAs have been effective and indispensable components in multistage solar distillation devices.

Moreover, Chen's group demonstrated another new‐strategy solar steam generation device using a couple of SSA and thermal emitter.^[^ ^176^ ^]^ The penetration depth of visible light in water is as large as 40 m (for the light of 0.5 µm), so the thick water cannot be heated a lot directly under sunlight. However, IR light with wavelengths above 4 µm is easily absorbed by water and the penetration depth is only less than 20 µm, indicating strong heat localization in an ultrathin water layer. Based on this principle, the contactless solar steam generation is created. Specifically, the device with an SSA can effectively harvest the solar energy and convert it into heat, which is re‐emitted in the form of LWIR light by a selective thermal emitter and directly absorbed by the topmost water layer, generating high‐temperature steam of 135 °C under 1.5 suns. The clever design partially learned from the configuration of solar thermophotovoltaics but the final outputs are different.^[^ ^64^ ^]^

### 6.2 Recently Developed Lab‐Scale SSAs

Besides the implementation of commercial SSAs on ISSG systems, lab‐scale SSAs developed by some research groups were also used to generate steam and freshwater. A CuO/Cu semi‐SSA was developed by chemical etching the Cu substrates and forming CuO nanoparticles. Near‐perfect absorption of >95% from 250 to 800 nm was achieved in the absorber, accompanied by strong reflection in the mid‐IR range.^[^ ^177^ ^]^ The CuO/Cu SSA produces vapor of 67 °C under one sun, which was 5 °C higher than the temperature of vapor generated by a CuO/Cu absorber with poorer spectral selectivity. The relatively narrow absorption band of this SSA limited the vapor temperatures <100 °C. Huang et al. fabricated a C–Au–TiO_2_ cermet SSA by sol–gel methods, which offered ≈90% absorption in the wavelength range of 250–1000 nm and ultralow emission <5% from 8 to 20 µm. The evaporation rate of the SSA floating on water is 1.04 kg m^−2^ h^−1^ with a solar‐thermal efficiency of 64%.^[^ ^31^ ^]^ More recently, Wang et al. fabricated a Ni‐coated anodic aluminum oxide (AAO) SSA with an $\bar{\alpha }$ of 83.2% and an ${\bar{\varepsilon }}_{100{\;}^{\circ }\text{C}}$ of 21.5% for ISSG.^[^ ^178^ ^]^ The SSA offered notable evaporation rates of 1.10 and 4.22 kg m^−2^ h^−1^ under 1 and 4 sun illumination, respectively, superior to those of nonselective graphite absorber ($\bar{\alpha }$ = ${\bar{\varepsilon }}_{100{\;}^{\circ }\text{C}}$ ≈ 95%).^[^ ^179^ ^]^ A graphene–Cu PhC‐based SSA was prepared by laser writing and self‐assembly graphene coating, providing both an $\bar{\alpha }$ of 91.9% and an ultralow $\bar{\varepsilon }$ of 3.8%. The average water evaporation rate of the SSA is measured to be 1.5 kg m^−2^ h^−1^, close to the theoretical limit of the evaporation rate under 1 sun.^[^ ^120^ ^]^ The primary concern of the usage of commercial or recently developed SSAs in ISSG systems is their solid structure without penetrative pores as water channels, which indicates additional channels need to be drilled for water supply and vapor escape. The postdrilled water channels generally have a relatively large size and the limited number that cannot effectively supply water to cover the whole SSA surface and fully utilize the evaporation area, thereby limiting the evaporation rate.

**Figure 22:**
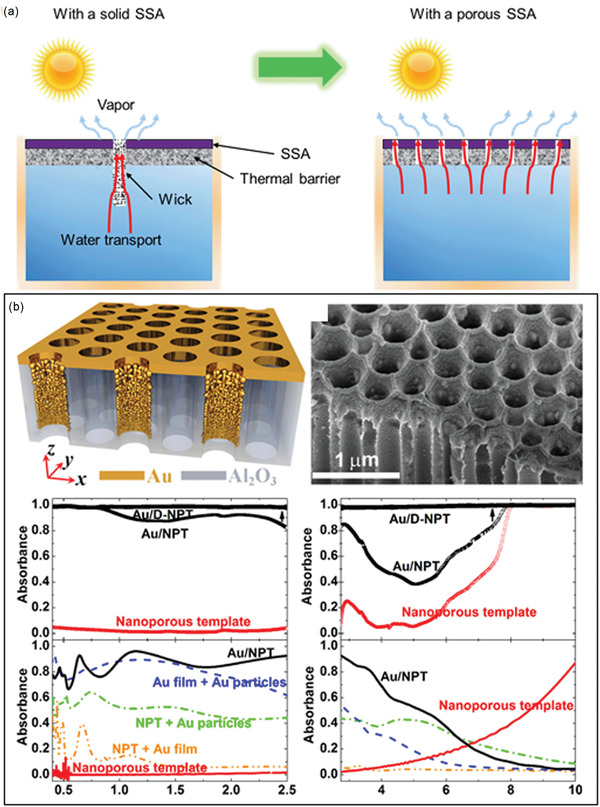
a) Schematic of current ISSG systems with a solid SSA assisted by wick for water transport, and a new ISSG system with a porous SSA that has self‐contained water channels. b) Schematic, SEM, and measured and simulated absorptance spectra of plasmonic absorbers with self‐contained water channels for ISSG. Reproduced according to the terms of the CC‐BY license.^[^ ^78^ ^]^ Copyright 2016, the authors.

SSAs with self‐contained water channels are more promising due to their multiple functions of solar harvesting, reduced radiation heat loss, and whole‐surface water supply (**Figure** **22**a). However, few demonstrations of using such porous SSAs in ISSG have been reported so far. In 2016, Zhou et al. reported self‐assembled plasmonic absorbers that fabricated by short‐term sputtering Al and Au nanoparticles on AAO membranes without solid substrates (Figure 22b).^[^ ^9^, ^78^, ^180^ ^]^ The well‐aligned pores of the AAO template acted as water channels. High solar absorptance $\bar{\alpha }$ of >95% is achieved by capturing the full‐spectrum sunlight.^[^ ^9^, ^78^ ^]^ The solar‐vapor efficiency of the plasmonic absorbers is around 58% and 90% under 1 and 4 sun illumination, respectively.^[^ ^9^, ^78^ ^]^ Intensive attention has been paid to these works because of their simple but effective designs for interfacial steam generation, but the spectral selectivity of these plasmonic absorbers has been neglected. The cut‐off wavelength of these SSAs can be tuned by changing the pore size of the AAO membranes and the depth of pores coated with nanoparticles.^[^ ^78^, ^180^ ^]^ However, the absorber with spectral selectivity (Au/NPT) shows a lower evaporation rate than the absorber without spectral selectivity (Au/D‐NPT) for two reasons regarding optical performances.^[^ ^78^ ^]^ First, the $\bar{\alpha }$ of the Au/NPT selective absorber (90%) was lower than that of the Au/D‐NPT (99%). Second, the absorption of the Au/NPT decreased in the wavelength range of 2–8 µm but increased to 99% at the peak wavelengths of blackbody radiation (8–13 µm for 30 °C blackbodies), so the spectrally averaged emissivity $\bar{\varepsilon }$ still was large. Great improvements can be expected by optimizing the structure parameters to achieve better solar‐thermal conversion performance.

We believe that spectral selectivity has contributed to the high efficiency of more reported solar absorbers more or less, especially those metal‐based absorbers. Continuous metal films such as Au and Ag with a small thickness of only 30 nm can offer >95% reflection in the mid‐IR region (>2.5 µm). Moreover, nanoscale pores (<500 nm) in the metal films will not significantly reduce their IR reflection due to the size mismatch.

## 7 Current Applications of Spectrally Selective IR Emitters on Water Harvesting

### 7.1 Sorbent‐Based Radiative Cooling Systems

**Figure 23:**
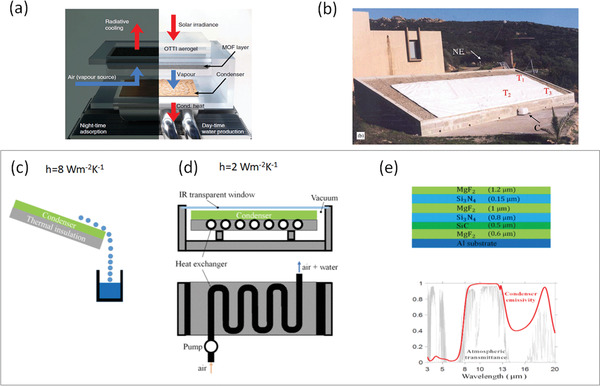
Schematics of various AWH devices. a) Hygroscopic sorbent‐based radiative cooling system. Reproduced with permission.^[^ ^40^ ^]^ Copyright 2018, Nature Publishing Group. b) Nighttime passive radiative condenser. Reproduced with permission.^[^ ^81^ ^]^ Copyright 2006, Elsevier. c) Daytime passive radiative condenser (typical rooftop design), d) daytime passive radiative condenser (optimized vacuum design), and e) structure and emissivity of the emitters in this daytime passive radiative condenser. (c–e) Reproduced with permission.^[^ ^84^ ^]^ Copyright 2020, Taylor & Francis.

Sorbent‐based AWH consists of the following three processes: i) water vapor in the air is captured into a sorbent, ii) water vapor is released by heating, and iii) water vapor is condensed into the liquid state.^[^ ^181^ ^]^ The adsorption relies on the water affinity provided by the material properties and component‐level properties of the sorbent material, and it can happen at a relatively low RH environment.^[^ ^181^, ^182^ ^]^ While silica gels and zeolites have drawbacks of low adsorption capacity and high energy needed for desorption, MOF was developed to capture water vapor from the air in the very arid climate with RH below 40 at night.^[^ ^39^, ^40^ ^]^ In the daytime, the water vapor is released and condense, providing a water harvesting rate of about 0.21 L m^−2^ day^−1^ (i.e., 0.25 L day^−1^ for 1 kg of 5 mm thick MOF), which is limited by its long recovery time in this two‐process‐daily‐cycle. Such technology uses a Pyromark‐coated layer functioned as a passive radiative cooler to facilitate vapor capture, solar absorber to provide heating to release vapor, and thermoelectric cooler to condense the vapor (**Figure** **23**a).^[^ ^40^ ^]^ So strictly speaking, it is not yet an off‐grid technology at this moment, although it has such potential in the near future. Recently, some other sorbent‐based AWH were reported,^[^ ^41^, ^183^, ^184^ ^]^ which utilize solar energy and natural cooling during the desorption and condensation processes, and it is believed that water harvesting rate can be improved by an enhanced cooling where passive radiative cooling can be a promising passive and cost‐effective option.

### 7.2 Nighttime Passive Radiative Slope

As reported in a paper in 1996, condensation was observed on the cooling panel with pigmented foils of low‐density polyethylene and sub‐microscaled TiO_2_ and BaSO_4_, in Sweden and Tanzania during the few last hours before sunrise while humidity reached RH80‐85. Although the condensing rate was only 0.09 L m^−2^ night^−1^, it inspired further research on harvesting dew water from the atmosphere in arid places (where RH is below 40 in the late afternoon) with an inexpensive scalable setup.^[^ ^185^ ^]^ After optimized the slope's tilt angle as a 20°–30° to minimize natural convection effect and maximize the dew drop recovery by gravity,^[^ ^45^ ^]^ a year‐round study reported in 2006 demonstrated a dew condensation achieved in humid condition (RH80‐90) by polyethylene (PE) foil‐base condensing slopes.^[^ ^81^ ^]^ These PE‐based setups harvested more dew than its referenced PMMA‐based setting and showed better performance in higher relative humidity environment and winter season (Figure 23b). In another report by the same research group, condensation highly depended on weather conditions, with a maximum daily dew condensation of less than 0.5 L m^−2^ night^−1^ according to their yearly study.^[^ ^186^ ^]^ In another research in 2015, dew condensation has been achieved by a highly emissive black low‐density PE foil typically used as soil mulching in agriculture. Higher condensing rate happened at nighttime with higher air humidity and less wind speed, and its maximum observed dew yield was about 0.28 L m^−2^ night^−1^.^[^ ^187^ ^]^

### 7.3 Daytime Passive Radiative Condenser

With the development of daytime radiative cooling, a recent study in 2020 discussed the optimized thermal management for the daytime radiative condenser, by minimizing the parasitic heat gain. It stated that the convective coefficient can be considered as *h* = 8 W m^−2^ K^−1^ for a slope design in a tropical rooftop setup (Figure 23c); while *h* value can be reduced to 2 W m^−2^ K^−1^ for the design that condenses within pipes beneath the radiative cooling panel (Figure 23d). According to their calculation, a thermally optimized design with daytime radiative emitter consisting of six layers of MgF_2_, Si_3_N_4_, and SiC can condensate at a rate of 0.04 L m^−2^ h^−1^ at RH100 and linearly decrease to 0 L m^−2^ h^−1^ at RH72 (when *h* = 8 W m^−2^ K^−1^), and 0.03 L m^−2^ h^−1^ at RH100 and linearly decrease to 0 L m^−2^ h^−1^ at RH32 (when *h* = 2 W m^−2^ K^−1^).^[^ ^84^ ^]^ As a result, atmospheric water harvester by passive radiative cooling is a promising environmentally friendly technology to resolve the global water scarcity problem, in particular in those off‐grid regions.

### 7.4 Comparison of AWH Performance

**Figure 24:**
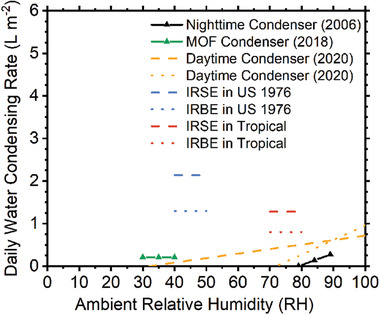
Comparison of daily atmospheric water condensing rate for different kinds of condensers, and calculated theoretical limits for ideal IRSEs and IRBEs. Solid lines represent for experiment data while dashed lines represent for simulation results. The calculation for the daytime condenser here is based on the hourly rate provided by literature, assuming the condensing rate is constant for the 24 h in this calculation.

A human body requires a minimum of 2 L water for drinking per day. As summarized in **Figure** **24**, AWH technologies show a quite satisfactory potential for real applications, e.g., a roof‐size condenser on top of a village house can provide a family's basic water requirement. Figure 24 is plotted on a daily basis for easy comparing sorbent‐based condensers that work in cyclic‐working mode with radiative condensers in a relatively continuous working state. The daily condensing rate of the nighttime condenser and MOF‐condenser are both from literature,^[^ ^40^, ^81^ ^]^ while the daily condensing rate of the daytime condenser is a rough estimation from the reported hourly rate, based on the assumption that the environmental conditions including ambient temperature and ambient RH are unchanged.^[^ ^84^ ^]^ Figure 24 only illustrates a rough comparison among the categories, due to different and complicated test conditions. However, it is also worthy to note that, due to the different working mechanisms, the sorbent‐based condenser and radiative condenser are applicable in different RH scenarios. Although the radiative condenser shows comparable performance at low ambient RH as a sorbent‐based AWH device, it can harvest water at a wider ambient RH range. As the daytime radiative cooling technology evolved from the nighttime one by adding strong solar reflection (>95%), daytime radiative condensers show greater potential in water condensation due to higher rates and longer working hours.

Figure 24 also shows that the ideal selective emitter (IRSE) has a much higher water condensing rate than the broadband emitter (IRBE) at a fixed environmental condition. For a given external environment and a device design with reduced parasitic heat gain, the emitter property is considered to play the most important role in the overall condensing efficiency. With the spectral selectivity *η_ε_* of merely around 1.45, this work of daytime‐radiative condenser was just a proof‐of‐concept study focus more on thermal optimization of the whole device,^[^ ^84^ ^]^ so it is believed that there is plenty of room for further improvement by optimizing spectral selectivity of the radiative material.

## 8 Conclusion and Outlook

### 8.1 Conclusion

We have presented a comprehensive review of the recent advances in solar selective absorbers for solar‐thermal energy conversion and IR selective emitters for passive radiative cooling, as well as their implementations on water production. According to the strategies used to achieve selective absorption, we have classified current selective absorbers/emitters into three main categories, intrinsic absorbers, optical cavities (including multilayer thin films and photonic crystals), and particle‐resonance absorbers (including particle dispersed composites and metamaterial absorbers). Intrinsic materials have the simplest design, but both their spectral selectivity and absorption/emission intensity are limited. Multilayer thin films based on interference effects and intrinsic materials offer better overall performance. Particle‐resonance absorbers have the potential to provide the best spectral selectivity and the strongest absorption intensity but with compromises in the structural complexity and fabrication challenges. Current applications of selective absorbers/emitters on the solar steam generation and radiative cooling enabled atmospheric water harvesting have also been introduced to motivate more research efforts in this promising area.

### 8.2 Challenges and Opportunities

Despite remarkable advances in these fields, there are still a lot of challenges in both the selective absorbers/emitters and their practical applications on water production.

#### 8.2.1 Tradeoffs among Optical Performance, Stability, and Structural Complexity

There are at least three tradeoffs between 1) the spectral selectivity and absorption/emission intensity, 2) the optical performance and thermal stability, and 3) the overall performance and structural complexity.

In the field of solar selective absorbers, the adoption of high‐loss materials such as W significantly enhances the absorption intensity and bandwidth but reduce the spectral selectivity due to the higher IR absorption than Au and Ag. For IR selective emitters, the tradeoff between the spectral selectivity and emission intensity is more remarkable, as summarized in Figure 20. Most of the current IR emitters offer satisfactory emission intensity but insufficient spectral selectivity.

In addition, the compromise between optical performance and thermal stability is common for solar selective absorbers, as shown in Figure 14. Photonic crystals embrace the highest tolerable temperatures, but solar‐thermal conversion efficiency for most of them is inferior to other classes of absorbers. Most of cermets and multilayer thin films with great solar‐thermal efficiency can only operate at temperatures <600 °C because the metal nanoparticles (in cermets) and nanofilms (in multilayer thin films) with ultrasmall sizes (≈10 nm) are bound to degrade at higher temperatures even in vacuum conditions. All‐ceramic solar selective absorbers show great potential in resolving these challenges.

From intrinsic materials to multilayer thin films and particle‐resonance absorbers/emitters, spectral selectivity, absorption/emission intensity, and therefore efficiency are dramatically improved; however, the optical structures become more and more complex, which brings challenges in large‐scale fabrication. Both perfect metamaterial absorbers and photonic crystals with promising performances generally need sophisticated lithographic methods to precisely control the nanoscale pattern sizes.

#### 8.2.2 Large‐Scale Deployment and Cost Reduction

Secondly, large‐scale deployment is limited by high fabrication costs of current superior selective absorbers/emitters. Except for some intrinsic materials, most of the selective absorbers/emitters based on nanophotonic structures have to be prepared by complicated high‐vacuum deposition methods or nanofabrication techniques in clean rooms, such as PVD, CVD, e‐beam lithography, and reaction ion‐etching. Since the optical properties of these nanostructures are extremely sensitive to the film thickness, nanoparticle size, and pattern feature size at the nanoscale level, it is quite challenging to manufacture high‐performance and robust absorbers/emitters (especially inorganic ones) using some low‐cost and scalable approaches, like solution‐based processes (spin coating, spray coating, dip coating, painting, etc.). However, significant cost reductions can be expected by developing solution‐processed selective absorbers/emitters.^[^ ^188^, ^189^, ^190^, ^191^ ^]^

#### 8.2.3 Holistic Connection for Selective Absorber/Emitter Studies

Third, there lacks a holistic connection for selective absorber/emitters operating in different spectra, which is also one of the motivations of this review. A broad variety of selective absorbers/emitters have been demonstrated for specific applications in different wavelength ranges. For instance, solar selective absorbers target for solar‐thermal energy conversion, UV and NIR selective absorbers for transparent solar cells, and IR selective emitters target for passive radiative cooling. These absorbers/emitters in different wavelength ranges shared similar features in configurations or absorption origins. However, connections among these absorbers/emitters have not been well established, which are beneficial for understanding them in a big picture and finding research gaps through mutual learning. Positive examples are perfect metamaterial absorbers, which consist of metal nanopatterns, dielectric spacers, and metal ground layers that have been extended from microwave to THz, IR, and visible regions.

#### 8.2.4 Practical Applications in Steam Generation and Water Production

Last but not least, the implementations of selective absorbers/emitters in interfacial solar steam generation and radiative cooling‐enabled atmospheric water harvesting are still in their early stages. In those interfacial solar steam generation devices with selective absorbers, high‐temperature steam has been generated under unconcentrated sunlight, but the evaporation rates are still relatively low. One of the main reasons is that solid selective absorbers cannot supply enough water to the surface and make full use of the evaporation area. Therefore, new‐concept solar selective absorbers with self‐contained penetrative pores are desired to improve the water and steam transport. However, these pores may lead to a decrease in solar absorption and an increase in thermal emission, so the distribution and size of pores are critical features. Besides, the contactless solar steam generation that also employs solar selective absorbers emerges as a very promising technology. On the other hand, with the recent rapid development of the daytime‐radiative cooling technology, it is believed that there can be novel water condenser designs with IR selective emitters to harvest a significant amount of water even in severe environments. It also requires a deeper investigation of theoretical study and the tailor‐design of emitter materials as well as device construction according to the specifically targeted application scenario. Device optimization to reduce parasitic heat gain is also of great need, to ensure the design can harvest atmospheric water at a satisfying amount in reality. There is still some way to go from fundamental scientific investigation to the engineering optimization for ready‐to‐market applications.

Challenges and opportunities coexist. If we can seize these opportunities by making better use of selective absorbers/emitters, we can efficiently utilize the sun (as a heat source) and outer space (as a heat sink) to facilitate the harvesting of freshwater that appears in nature, a bright and sustainable future is beckoning.

## Conflict of Interest

The authors declare no conflict of interest.
